# Socioeconomic status, non-communicable disease risk factors, and walking speed in older adults: multi-cohort population based study

**DOI:** 10.1136/bmj.k1046

**Published:** 2018-03-23

**Authors:** Silvia Stringhini, Cristian Carmeli, Markus Jokela, Mauricio Avendaño, Cathal McCrory, Angelo d’Errico, Murielle Bochud, Henrique Barros, Giuseppe Costa, Marc Chadeau-Hyam, Cyrille Delpierre, Martina Gandini, Silvia Fraga, Marcel Goldberg, Graham G Giles, Camille Lassale, Rose Anne Kenny, Michelle Kelly-Irving, Fred Paccaud, Richard Layte, Peter Muennig, Michael G Marmot, Ana Isabel Ribeiro, Gianluca Severi, Andrew Steptoe, Martin J Shipley, Marie Zins, Johan P Mackenbach, Paolo Vineis, Mika Kivimäki

**Affiliations:** 1 Institute of Social and Preventive Medicine, Lausanne University Hospital, Biopôle 2-Route de la Corniche 10, 1010 Switzerland; 2 Department of Psychology and Logopedics, Faculty of Medicine, University of Helsinki, Helsinki, Finland; 3 Department of Global Health and Social Medicine, King’s College London, London, UK; 4 Harvard T.H. Chan School of Public Health, Boston MA, USA; 5 The Irish Longitudinal Study on Ageing (TILDA), Trinity College Dublin, Dublin, Ireland; 6 Epidemiology Unit, ASL TO3 Piedmont Region, Grugliasco (TO), Italy; 7 EPIUnit-Institute of Public Health, University of Porto, Porto, Portugal; 8 Department of Clinical Epidemiology, Predictive Medicine and Public Health, University of Porto Medical School, Porto, Portugal; 9 MRC-PHE Centre for Environment and Health, School of Public Health, Department of Epidemiology and Biostatistics, Imperial College London, London, UK; 10 INSERM, UMR1027, Toulouse, France, and Université Toulouse III Paul-Sabatier, Toulouse, France; 11 Population-based Epidemiological Cohorts Unit, INSERM UMS 11, Villejuif, France, and Paris Descartes University, Paris, France; 12 Cancer Epidemiology Centre, Cancer Council Victoria, Melbourne, VIC, Australia; 13 University College London, Department of Epidemiology and Public Health, London, UK; 14 Department of Sociology, Trinity College Dublin, Dublin, Ireland; 15 Global Research Analytics for Population Health, Health Policy and Management, Columbia University, New York, NY, USA; 16 CESP, Inserm U1018, Université Paris-Saclay, Villejuif, France; 17 Human Genetics Foundation (HuGeF), Turin, Italy; 18 Department of Public Health, Erasmus University Medical Center, Rotterdam, Netherlands; 19 Clinicum, Faculty of Medicine, University of Helsinki, Finland

## Abstract

**Objective:**

To assess the association of low socioeconomic status and risk factors for non-communicable diseases (diabetes, high alcohol intake, high blood pressure, obesity, physical inactivity, smoking) with loss of physical functioning at older ages.

**Design:**

Multi-cohort population based study.

**Setting:**

37 cohort studies from 24 countries in Europe, the United States, Latin America, Africa, and Asia, 1990-2017.

**Participants:**

109 107 men and women aged 45-90 years.

**Main outcome measure:**

Physical functioning assessed using the walking speed test, a valid index of overall functional capacity. Years of functioning lost was computed as a metric to quantify the difference in walking speed between those exposed and unexposed to low socioeconomic status and risk factors.

**Results:**

According to mixed model estimations, men aged 60 and of low socioeconomic status had the same walking speed as men aged 66.6 of high socioeconomic status (years of functioning lost 6.6 years, 95% confidence interval 5.0 to 9.4). The years of functioning lost for women were 4.6 (3.6 to 6.2). In men and women, respectively, 5.7 (4.4 to 8.1) and 5.4 (4.3 to 7.3) years of functioning were lost by age 60 due to insufficient physical activity, 5.1 (3.9 to 7.0) and 7.5 (6.1 to 9.5) due to obesity, 2.3 (1.6 to 3.4) and 3.0 (2.3 to 4.0) due to hypertension, 5.6 (4.2 to 8.0) and 6.3 (4.9 to 8.4) due to diabetes, and 3.0 (2.2 to 4.3) and 0.7 (0.1 to 1.5) due to tobacco use. In analyses restricted to high income countries, the number of years of functioning lost attributable to low socioeconomic status by age 60 was 8.0 (5.7 to 13.1) for men and 5.4 (4.0 to 8.0) for women, whereas in low and middle income countries it was 2.6 (0.2 to 6.8) for men and 2.7 (1.0 to 5.5) for women. Within high income countries, the number of years of functioning lost attributable to low socioeconomic status by age 60 was greater in the United States than in Europe. Physical functioning continued to decline as a function of unfavourable risk factors between ages 60 and 85. Years of functioning lost were greater than years of life lost due to low socioeconomic status and non-communicable disease risk factors.

**Conclusions:**

The independent association between socioeconomic status and physical functioning in old age is comparable in strength and consistency with those for established non-communicable disease risk factors. The results of this study suggest that tackling all these risk factors might substantially increase life years spent in good physical functioning.

## Introduction

In the context of a rapidly aging world population, global health strategies have considered healthy aging as a public health priority.^1^ ^2^ The World Health Organization Global Action Plan for the Prevention and Control of Non-Communicable Diseases, for example, has set member states a goal by 2025 to reduce premature mortality from chronic diseases by 25%.^3^ This is to be achieved by targeting high alcohol consumption, insufficient physical activity, tobacco use, high blood pressure, excess salt intake, diabetes, and obesity. Recent studies suggest that targeting adverse socioeconomic circumstances or low socioeconomic status in addition to these risk factors might lead to additional gains in longevity.^4^ ^5^ Socioeconomic status is a sociological construct referring to an individual’s relative position in the social hierarchy, as measured by indicators such as occupational group, educational attainment, level of income and wealth, and place of residence.

In addition to prevention of age related morbidity and premature mortality, minimising the time that people spend living with disability and dependence is a major public health challenge. Although several investigations have found socioeconomic status and risk factors for non-communicable diseases to predict mobility and disability,^6^ ^7^ ^8^ ^9^ ^10^ ^11^ ^12^ ^13^ ^14^ ^15^ ^16^ ^17^ ^18^ whether socioeconomic status has a similar predictive value for functioning as established non-communicable disease risk factors has not yet been explored. Using national data across different contexts and regions to examine the consistency of the associations of socioeconomic status and non-communicable risk factors with physical functioning and mortality within a single analytic setting would be useful because of implications for priority setting of risk factors as well as planning health and social policies. We hypothesised that socioeconomic status is related to greater absolute differences in physical functioning at older ages than younger ages as is the case for established non-communicable disease risk factors.

We compared the association between socioeconomic status and physical functioning with those between established non-communicable disease risk factors and physical functioning from early to advanced age. To obtain reliable and generalisable estimates, we pooled harmonised individual level data from 37 studies spanning 24 countries from Europe, the United States, Europe, Latin America, Africa, and Asia. We used walking speed as a measure of physical functioning because it is a simple and sensitive indicator of overall functional capacity.^19^ As walking is a complex task requiring energy, balance, movement control, and coordination of the musculoskeletal, nervous, respiratory, and cardiovascular systems,^19^ disturbance or deterioration in any of these modalities tends to affect the speed of walking.^20^ ^21^ Furthermore, walking speed declines with age and predicts functional limitations, hospital admission, clinical and subclinical disease, comorbidities, frailty, admission to residential care, and mortality,^19^ ^22^ ^23^ ^24^ ^25^ ^26^ ^27^ ^28^ even in old age.^29^

## Methods

### Study populations

The present study is part of an EC Horizon 2020 consortium, the LIFEPATH project. In the present analysis we included a total of 37 studies comprising 109 107 men and women aged 45 to 90 years from 24 WHO member countries: UK, France, USA, Mexico, China, Ghana, India, Russia, South Africa, Costa Rica, Taiwan, Ireland, Austria, Belgium, Denmark, Germany, Greece, Israel, Italy, the Netherlands, Portugal, Spain, Sweden, and Switzerland. We chose the 45-90 age range as few data were available for younger or older age groups and the meaning of walking speed in the age group 30-40 years is unclear.

Data were collected between 1990 and 2017. All studies included data on socioeconomic status and walking speed. We excluded people who used walking aids because walking speed was used as a proxy measure of overall functioning, and the assessment is not reliable among participants using walking aids. The relevant local or national ethics committees approved each study, and participants gave informed consent to participate. Six datasets were part of the LIFEPATH Consortium (the GAZEL, ELSA, WHITEHALL II, CONSTANCES, TILDA, and EPIPORTO studies), six were part of the WHO Study on global AGEing and adult health (SAGE), 12 were from the Survey of Health, Ageing and Retirement in Europe (SHARE), four were from the InterUniversity Consortium for Political and Social Research (MIDUS, HEPESE, SEBAS, and NSHAP), one was from the Health and Retirement Study (HRS), two were from the Wisconsin Longitudinal Study (WLSG and WLSS), three were from the National Health and Nutrition Examination Survey (NHANES III, NHANES 1999, and NHANES 2001), two were from the Costa Rican Longevity and Health Ageing Study (CRELES Pre 1945 and CRELES RC), and one was from the Health and Ageing Study in Africa: A Longitudinal Study of an INDEPTH Community in South Africa (HAALSI). The appendix provides details of the study design, participants, and cohort descriptions.

### Risk factors

In the LIFEPATH Consortium, we used a predefined harmonised definition of socioeconomic status, as described in previous papers.^30^ ^31^ We used information on several social indicators available in the participating cohort studies—education, own and father’s occupational class, and income. For adults, we assigned socioeconomic status to cohort members using information on the last known occupational title at study enrolment. To obtain a harmonised measure of occupational class across the study cohorts, we classified occupations according to the European socioeconomic classification, which is a classification based on the nature of employment relationships.^32^ We predefined and harmonised such data across the study cohorts before analyses.^4^ We categorised occupational class into high (higher professionals and managers, higher clerical, services, and sales workers (European socioeconomic class 1-3)), intermediate (small employers and self employed, farmers, lower supervisors and technicians, class 4-6), or low socioeconomic status (lower clerical, services, and sales workers, skilled workers, semi-skilled and unskilled workers, class 7-9).^33^ Given the large proportion of participants (>50%) with no formal occupation, in the case of HAALSI, we used a measure of wealth categorised in thirds.

Self reported smoking was categorised into current, former, and never. Alcohol consumption was measured in alcohol units weekly, and we categorised participants as non-drinkers (0 units/week), moderate drinkers (1-21 units/week for men, 1-14 units/week for women), or harmful drinkers (>21 units/week for men, >14 units/week for women). Leisure physical activity was measured with different questions in each study, making it impossible to derive a comprehensive definition. As a result, we dichotomised leisure physical activity as sufficient or insufficient in each study using study specific thresholds (see supplementary appendix 2). Height and weight were measured using standard procedures; body mass index (BMI) was calculated as kg/m^2^ and categorised as normal (18.5 to <25), overweight (25 to <30), or obese (≥30), with those who were underweight (<18.5) excluded from the BMI analyses. Hypertension was defined as the presence of at least one of systolic blood pressure ≥140 mm Hg, diastolic blood pressure ≥90 mm Hg, current antihypertensive treatment, or self reported hypertension. Diabetes was defined as the presence of at least one of fasting glucose concentration ≥7 mmol/L, post-load glucose concentration >11.1 mmol/L at two hours, glycated haemoglobin A1c level ≥6.5%, or self reported diabetes.

### Covariates

We considered age, height, and year of birth (five year intervals) as potential confounders. Height is a correlate of walking speed, as longer legs are associated with higher walking speed (allometric dependence of walking speed). In addition, height is a marker of childhood health or disease, which can affect both walking speed in adulthood and functioning in old age. In supplementary models, we also controlled for ethnic origin and baseline health status.

### Walking speed

In all cohorts, participants were instructed to walk at their usual pace. The setup differed among cohorts, (see supplementary table S1 for details). The distance walked varied from 8 feet (2.4 m) in ELSA, WHITEHALL II, NHANES III, and HEPESE to 15.24 m in MIDUS. Walking time was recorded with photoelectric devices for some cohorts and manually by an interviewer for others. In all studies, time walked was averaged across multiple trials and was then converted into an overall measure of speed, expressed as metres travelled per second (m/s). Walking speed was assessed at the same time as participant risk factors.

### Mortality

In a subset of 24 cohorts (table 1), 83 783 participants were linked to national mortality registries that provided information about vital status. We set the baseline for mortality analysis at the same wave in which gait speed measures were obtained. Mean mortality follow-up ranged between 2.9 years (MIDUS) and 21.4 years (NHANES 3), with a mean of 8.1 years (SD 5.2 years) across cohorts.

**Table 1:** Characteristics of study populations. Values with a slash represent men and women, respectively

Study	Baseline for this study	Country	No of participants	Mean (SD) age at baseline (years)	Walking distance (m)	Mean walking speed (m/s)	Mean mortality follow-up (years)
GAZEL	2010	France	1979/603	64.5 (2.8)/61.7 (4.0)	3	1.18/1.15	6.6/6.6
ELSA	2004-05	UK, England	2104/2545	70.1 (7.2)/70.4 (7.6)	2.44	0.91/0.84	7.3/7.6
WHITEHALL II	2002-04	UK, London	4384/1809	61.0 (5.9)/61.4 (6.0)	2.44	1.28/1.13	9.1/9.2
NHANES III	1990-93	USA	2277/2184	71.6 (8.0)/71.9 (8.1)	2.44	0.75/0.70	9.9/11.2
NHANES 1999	1999	USA	882/801	66.3 (9.8)/65.7 (10.1)	6.1	0.97/0.94	9.8/10.4
NHANES 2001	2001	USA	925/871	65.3 (10.2)/65.5 (10.3)	6.1	1.03/1.0	8.8/9.2
HRS	2006-09	USA	2685/3110	72.4 (6.3)/72.0 (6.5)	5	1.58/1.48	6.3/6.5
MIDUS	2004-05	USA	154/154	52.6 (6.7)/52.2 (6.2)	15.24	1.12/1.08	3.0/3.0
WLSG	2010-12	USA, Wisconsin	2519/2717	71.3 (0.9)/71.2 (0.9)	2.5	1.01/0.95	3.4/3.3
WLSS	2010-12	USA, Wisconsin	1333/1465	68.7 (6.7)/69.0 (6.6)	2.5	1.02/0.96	3.3/3.3
CONSTANCES	2012	France	13 593/14 819	57.9 (7.1)/57.4 (7.0)	3	1.29/1.25	None
CRELES-RC	2010	Costa Rica	1196/1766	61.0 (5.3)/58.7 (4.3)	3	1.04/0.98	None
CRELES Pre 1945	2005	Costa Rica	901/702	73.6 (7.6)/72.4 (8.0)	3	0.69/0.62	3.0/3.1
HAALSI	2014-15	South Africa, Agincourt	1691/1955	63.4 (10.6)/62.5 (10.8)	2.5	0.63/0.58	None
HEPESE	1993-94	USA, Mexican Americans	966/723	72.6 (5.8)/72.2 (5.5)	2.44	0.46/0.43	5.4/5.9
SEBAS	2006	Taiwan	514/331	66.2 (9.5)/63.3 (8.8)	3	0.86/0.80	None
NSHAP	2010-11	USA	1389/1626	72.7 (7.2)/71.4 (8.1)	3	0.70/0.68	None
SAGE China	2008	China	4652/4786	62.3 (9.4)/61.8 (9.4)	4	1.02/0.96	None
SAGE Ghana	2008	Ghana	2020/1804	61.7 (10.0)/63.2 (10.2)	4	0.81/0.71	None
SAGE India	2008	India	3150/1661	60.7 (9.0)/58.4 (8.9)	4	0.89/0.81	None
SAGE Mexico	2008	Mexico	502/370	65.5 (9.0)/64.5 (9.3)	4	0.88/0.78	None
SAGE Russia	2008	Russia	884/1570	61.4 (9.1)/63.3 (9.9)	4	0.77/0.70	None
SAGE South Africa	2008	South Africa	901/1159	61.5 (9.0)/62.1 (9.5)	4	0.85/0.77	None
SHARE Austria	2004	Austria	42/48	76.0 (8.8)/77.1 (8.2)	5	1.36/1.33	6.8/7.1
SHARE Belgium	2004-05	Belgium	170/162	78.6 (6.3)/77.6 (7.1)	5	1.47/1.26	6.0/6.7
SHARE Denmark	2004	Denmark	84/105	77.4 (9.1)/77.3 (8.5)	5	1.62/1.42	6.7/7.4
SHARE France	2004-05	France	141/177	77.6 (7.1)/78.3 (7.3)	5	1.33/1.20	6.2/6.8
SHARE Germany	2004	Germany	91/100	76.5 (7.1)/78.0 (6.3)	5	1.37/1.23	5.9/6.3
SHARE Greece	2004-05	Greece	83/69	78.7 (6.8)/76.1 (8.8)	5	1.14/1.07	4.5/4.5
SHARE Israel	2005-06	Israel	71/49	78.5 (5.8)/76.8 (7.6)	5	1.45/1.27	6.5/7.2
SHARE Italy	2004	Italy	85/76	75.8 (7.4)/74.1 (9.3)	5	1.28/1.02	7.1/7.5
SHARE Netherlands	2004	Netherlands	124/107	77.7 (7.7)/76.5 (8.5)	5	1.53/1.46	5.6/7.5
SHARE Spain	2004	Spain	118/115	77.4 (7.6)/75.0 (9.3)	5	1.26/1.08	7.2/7.6
SHARE Sweden	2004-05	Sweden	129/141	79.5 (5.7)/79.5 (5.5)	5	1.57/1.43	6.6/7.3
SHARE Switzerland	2004	Switzerland	43/56	80.7 (4.3)/79.9 (6.2)	5	1.66/1.45	5.6/7.1
TILDA	2009-11	Ireland	2149/2638	62.0 (8.4)/60.5 (8.4)	4.88	1.38/1.36	None
EPIPORTO	2016-17	Portugal	324/478	65.7 (10.3)/65.1 (9.4)	7.62	1.74/1.39	None

### Statistical analysis

#### Walking speed and age

We used a generalised additive mixed model (GAMM)^34^ to estimate walking speed, with age and height as fixed effect predictors and study as random effect at the intercept and age slope. GAMM is a semi-parametric model that uses a family of splines to obtain a smooth representation of the dependence of walking speed with age. The algorithm implemented in the R package gamm4 automatically selected the number of knots in the splines.^35^ We computed 95% confidence intervals from the uncertainty of the estimated smoothing function.

#### Years of functioning lost

We computed the number of years of functioning lost from the mixed model predictions of walking speed along with age. The mixed model of walking speed included a random effect of study at the intercept and age slope. Fixed effects included age, age^2^, height, year of birth, distances walked, the risk factor under study (minimally adjusted models) or all risk factors (mutually adjusted models), and an interaction term between age and the risk factor. The structure of the models was determined through likelihood ratio tests.

Confidence intervals for years of functioning lost were determined through 5000 bootstrap samples, applying a model based parametric bootstrap method.^36^ For all examined risk factors, we computed the years of functioning lost associated with exposure by predicting the chronological age of the unexposed group equivalent to the walking speed at age 60 (or 85) of the exposed group. Years of functioning lost was then obtained as (60 (or 85) age reference). This method allows years of functioning lost at a given age to be calculated retrospectively, as opposed to the classic years of life lost calculated prospectively.

#### Years of life lost

Years of life lost were used as a secondary outcome, and we compared this with years of functioning lost, our primary outcome. We calculated years of life lost as the difference between the areas under the survival curves (from age 60 to 85) of the population exposed to a given risk factor compared with the unexposed reference population. Survival curves were estimated using Kaplan-Meier adjusted curves, conditional on survival to age 60 years. We ran a shared frailty Cox model^19^ with age as time scale, stratified by the levels of the given risk factor and year of birth as covariate (for minimally adjusted models) or year of birth and the remaining risk factors as covariates (mutually adjusted models). The shared frailty variable was introduced to account for study effect.^19^

### Patient involvement

No patients were involved in setting the research question or the outcome measures, nor were they involved in developing plans for recruitment, design, or implementation of the study. No patients were asked to advise on interpretation or writing up of results. There are no plans to disseminate the results of the research to study participants or the relevant patient community.

## Results

**Fig 1:**
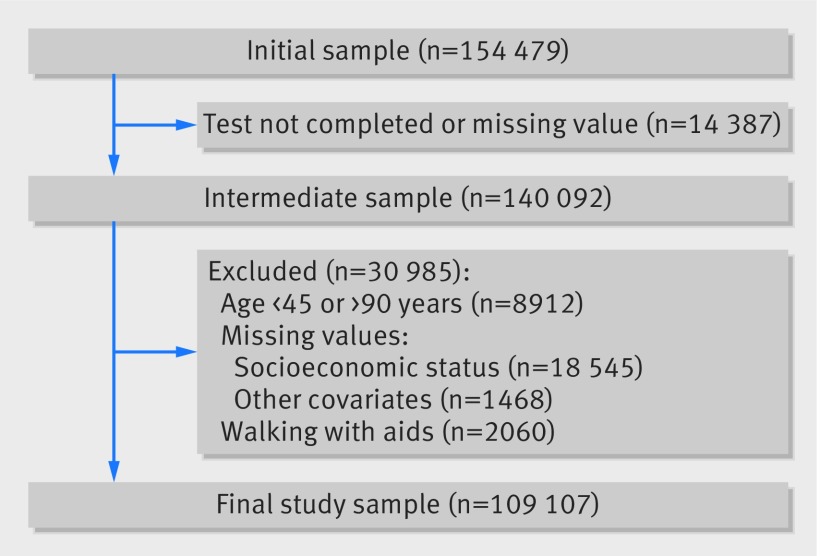
Flow diagram of participants included in study

Thirty seven studies from 24 countries were included. Of 140 092 participants with data on walking speed, we excluded 30 985 (8912 were outside the studied age range (45-90 years), 18 545 (14.1%) lacked data on socioeconomic status, 1468 lacked data on one or more of the covariates, and 2060 (1.9%) used a walking aid). This left 109 107 participants for analysis (fig 1). Compared with those included in the analyses, the excluded participants were younger (58.1 *v* 63.7 years, this is because we set 45 years as the minimum age), of a lower socioeconomic status (36.7% *v* 32.2%), and more likely to be women (64.8% *v* 49.4%). They were also more likely to have a normal BMI (46.8% *v* 41.0%), abstain from alcohol (64.3% *v* 42.9%), be physically inactive (32.4% *v* 29.4%), have hypertension (59.4% *v* 55.2%), and smoke (22.1% *v* 15.2%).

Of the 109 107 participants, 14 368 (13.2%) were aged 75-90 and 41 656 (38.2%) were younger than 60. The mean age for men was 63.9 (SD 9.4) and for women was 63.6 (SD 9.8); 49.4% were women **(** table 1). About one third of participants were in or had been in a low occupational class; 35.4% of men and 24.8% of women were in or had been in a high occupational class.

**Fig 2:**
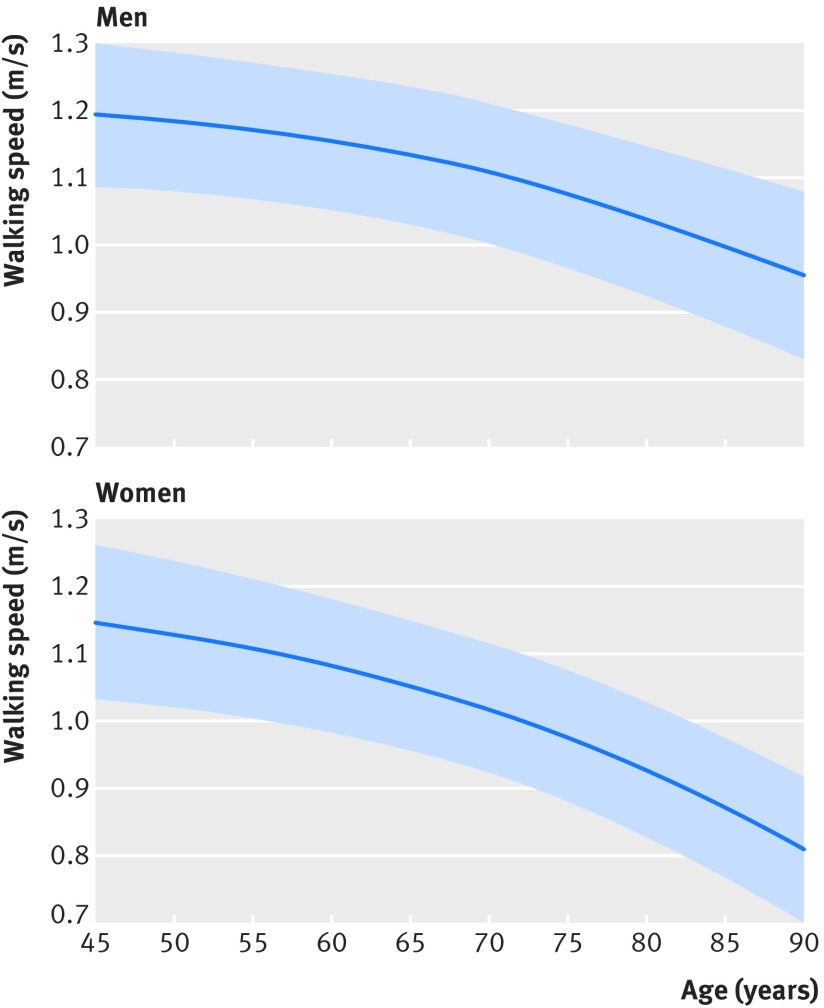
Walking speed as function of age in men and women

Figure 2 shows the age related decline in walking speed in 55 255 men and 53 852 women predicted using the GAMM model, conditioned to the average height of men and women. For men, walking speed declined from 1.19 m/s (95% confidence interval 1.08 to 1.30) at age 45 to 0.95 m/s (0.83 to 1.08) at age 90. For women, the decline was from 1.15 m/s (1.03 to 1.26) at age 45 to 0.81 m/s (0.70 to 0.92) at age 90. Age related decline in walking speed was not linear but accelerated after the ages of 65-70 for both men and women.

### Years of functioning lost by age 60 and 85

**Fig 3:**
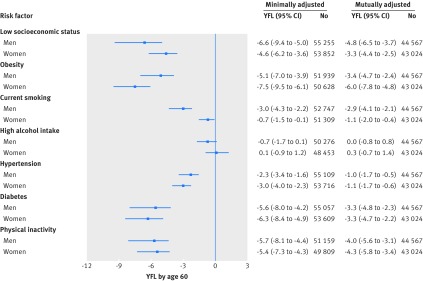
Years of functioning lost (YFL) by age 60 due to suboptimal risk factors. Minimally adjusted models were only adjusted for age, age^2^, height, year of birth, and distances walked; in mutually adjusted models, socioeconomic status and non-communicable diseases risk factors are mutually adjusted

Figure 3 shows the years of functioning lost by age 60 due to exposure to suboptimal risk factors, as measured by differences in walking speed. A 60 year old man of low socioeconomic status had the same walking speed as a 66.6 year old man of high socioeconomic status (95% confidence interval 5.0 to 9.4), whereas for women the difference was 4.6 (3.6 to 6.2) years. Years of functioning lost due to low socioeconomic status were comparable to years of functioning lost due to obesity, diabetes, and physical inactivity (range 5.1 to 5.6 for men and 5.4 to 7.5 for women), but they were greater than years lost due to tobacco smoking, hypertension, and high alcohol intake (range 0.7 to 3.0 for men and 3.0 to −0.1 for women). Results were comparable in analyses mutually controlling for all risk factors. Analyses were repeated using a two step approach including cohort specific analyses and pooling of cohort specific estimates using meta-analysis (see supplementary appendix 4). The approach yielded results similar to those found in the main analysis (see figure 3). Finally, we also estimated years of functioning lost by ages 50 and 70 (see supplementary appendix 3, figures S2 and S3); the pattern and magnitude of the associations between risk factor and years of functioning lost were comparable to those observed by age 60.

In analyses restricted to high income countries, years of functioning lost attributable to low socioeconomic status by age 60 were 8.0 (95% confidence interval 5.7 to 13.1) for men and 5.4 (4.0 to 8.0) for women (see supplementary appendix 3, figures S4 and S5). In low and middle income countries, the corresponding years of functioning lost were 2.6 (0.2 to 6.8) for men and 2.7 (1.0 to 5.5) for women. Within high income countries, years of functioning lost attributable to low socioeconomic status by age 60 were greater in the United States than in Europe (see supplementary appendix 3, figures S6 and S7): 19.7 (13.2 to 27.4) *v* 6.0 (3.8 to 12.1) for men and 15.8 (10.8 to 21.4) *v* 3.9 (2.6 to 7.2) for women. This was also the case for years of functioning lost attributable to other risk factors, in particular current smoking and high alcohol intake.

In a sensitivity analysis including participants who used walking aids, the number of years of functioning lost attributable to risk factors by age 60 did not materially differ from those reported in the main analysis (see supplementary appendix 3, figure S8). In supplementary multivariate analyses, adjusting for physical inactivity, ethnicity, or health status (see supplementary appendix 3, table S2-S4), or for the number of other risk factors to which participants were exposed (see supplementary appendix 3, table S6), years of functioning lost attributable to the risk factors by age 60 did not differ from those reported in the main analysis.

In an additional analysis where the maximum number of participants for each risk factor was used irrespective of the availability of socioeconomic status data (see supplementary appendix 3, table S5), the estimated years of functioning lost attributable to the non-communicable disease risk factors by age 60 did not differ from those reported in the main analysis. The association between obesity and walking speed was little changed at age 60 after inclusion of underweight participants in the reference group (see supplementary appendix 3, table S7).

**Fig 4:**
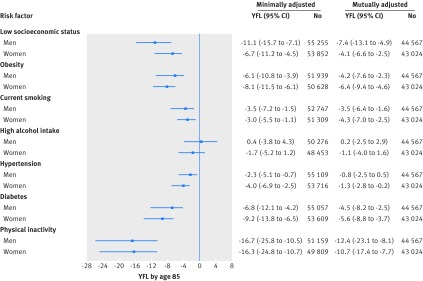
Years of functioning lost (YFL) by age 85 due to suboptimal risk factors. Minimally adjusted models were only adjusted for age, age^2^, height, year of birth, and distances walked; in mutually adjusted models, socioeconomic status and non-communicable diseases risk factors are mutually adjusted

Figure 4 shows years of functioning lost due to suboptimal risk factors by age 85. Walking speed continued to decrease as a function of risk factors between ages 60 and 85 (see supplementary appendix 3, figure S1), in particular for those of low versus high socioeconomic status (11.1 (95% confidence interval 7.1 to 15.7) years of functioning lost at age 85 for men and 6.7 (4.5 to 11.2) for women) and for those with insufficient versus sufficient physical activity (16.7 (10.5 to 25.8) years of functioning lost at age 85 for men and 16.3 (10.7 to 24.8) for women).

### Years of life lost between ages 60 and 85

**Fig 5:**
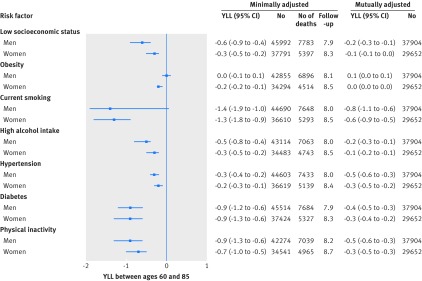
Years of life lost (YLL) between ages 60 and 85 due to suboptimal risk factors. Minimally adjusted models were only adjusted for year of birth; in mutually adjusted models, socioeconomic status and non-communicable diseases risk factors are mutually adjusted

Years of functioning lost were greater than years of life lost due to low socioeconomic status and non-communicable disease risk factors. Between ages 60 and 85 years, low socioeconomic status was associated with a loss of 0.6 (95% confidence interval 0.4 to 0.9) years of life for men and 0.3 (0.2 to 0.5) years of life for women (fig 5). Years of life lost due to suboptimal risk factors were similar for high alcohol intake (0.5 for men and 0.3 for women), greater for smoking (1.4 for men and 1.3 for women), diabetes (0.9), and physical inactivity (0.9 for men and 0.7 for women), and lower for hypertension and obesity.

## Discussion

Based on individual level data from 37 cohort studies and more than 109 000 adults, our analyses showed that adverse socioeconomic circumstances and exposure to major non-communicable disease risk factors are robustly associated with loss in physical functioning from early to a more advanced old age. By age 60, about six years of good physical functioning were lost due to poor socioeconomic circumstances. The years of functioning lost were comparable (ie, 5-7 years) for obesity, diabetes, and insufficient physical activity, and lower (between <1 and 3 years) for tobacco smoking, hypertension, and high alcohol intake. Exposure to adverse socioeconomic circumstances and the non-communicable disease risk factors continued to be associated with physical functioning after age 60, with increasing differences in functioning between exposed and unexposed groups at age 85. Years of functioning lost at older ages generally exceeded the years of life lost, suggesting that an exclusive focus on morbidity and mortality might lead to underestimation of the potential benefits of targeting poor socioeconomic circumstances and risk factors highlighted in global health strategies. The years of functioning lost due to these factors were particularly large in high income countries.

### Strengths and weaknesses of this study

We used walking speed to assess physical functional capacity. Previous research has shown that walking speed declines with age and is an independent predictor of survival, functional limitations, hospital admission, and cognitive decline.^19^ ^22^ ^23^ Compared with other measures of physical functioning, such as grip strength, walking speed requires the coordinated action of several different physical systems, including the nervous, musculoskeletal, and cardiopulmonary systems.^37^ Furthermore, walking speed is widely used in aging research as it is easily measurable in population surveys as well as in clinical settings. However, heterogeneity did exist in the measurement of walking speed between studies, which we partially accounted for through a mixed statistical modelling framework. Since walking speed is unlikely to capture all important aspects of “overall functional capacity,” further research with other indicators of physical functioning, such as grip strength and lung function would provide a useful comparison.

As our analyses relied on cross sectional data, these findings should be interpreted cautiously and should not be considered as causal estimates of the impact of socioeconomic status on health. For example, selection bias would attenuate observed associations if non-participation in studies was greater among those with poor functioning. Similarly, physical inactivity and obesity may be both a cause and a consequence of impaired mobility, potentially inflating the observed associations. However, our findings highlight the potential importance of socioeconomic status for physical functioning at older ages, and motivate further research to establish the causal nature of the observed associations. Randomised trials and quasi-experimental studies are required to evaluate the extent to which intervening on socioeconomic adversity and standard non-communicable disease risk factors might improve functional capacity in old age.

Occupational class was used as an indicator of socioeconomic status, as there were data available for this measure across the cohorts included in our study and occupational class was comparable between countries. Occupational class may not entirely reflect current socioeconomic conditions, particularly for older participants, although pensions and benefits after retirement are often a function of employment during active life. Since we compared the associations of low socioeconomic status with those of major non-communicable disease risk factors that are currently targeted by global health strategies, we did not include additional factors that might also affect functional capacity, such as psychosocial stress, social isolation, and exposure to environmental pollutants; the effects of these factors are an important topic of further studies.

Socioeconomic status and non-communicable disease risk factors were assessed using broad categorisations, and for some of the cohort studies only self reported data were available. Furthermore, the success in harmonisation varied between cohort studies depending on the availability of data. As a result misclassifications might exist, potentially underestimating or overestimating the associations between socioeconomic status, non-communicable disease risk factors, and physical functioning.

### Comparison with previous studies

Our study shows that the number of years of functioning lost due to adverse socioeconomic circumstances is comparable to or larger than the number of years lost due to major risk factors for chronic diseases, such as insufficient physical activity, diabetes, obesity, and tobacco consumption. Interestingly, this pattern persisted in models that controlled for all risk factors simultaneously. Risk factors, including socioeconomic adversity, tend to cluster in the same individuals, but our findings suggest that the association of low socioeconomic status with physical functioning is not attributable to non-communicable disease risk factors.

Years of functioning lost due to low socioeconomic status were greater in high income countries than in low to middle income countries. A potential explanation of this finding includes regional differences in the social patterning of major risk factors, such as physical inactivity, obesity, and diabetes. Unlike in high income countries, lower socioeconomic status is not always associated with a higher prevalence of these risk factors in low to middle income countries.^38^ ^39^ In addition, classifying individuals into occupational classes in low to middle income countries is difficult as large fractions of the population have informal jobs or employment contracts, and misclassification of occupational class may have attenuated the observed associations. Furthermore, life expectancy is shorter in low to middle income countries and this may lead to greater selection bias attenuating associations in studies from these countries.

Insufficient physical activity, obesity, and diabetes were strong independent predictors of loss of functional capacity (5-7 years of functioning lost), whereas the association of tobacco consumption, high alcohol intake, and hypertension with loss of functional capacity was weaker (0-3 years of functioning lost). The result was expected, given the adverse impact of insufficient physical activity, obesity, and diabetes on the musculoskeletal system. Our findings are consistent with studies showing that physical activity might improve physical performance^15^ ^40^ even at older ages,^41^ ^42^ ^43^ although part of the observed effect is also likely to be due to a reverse causation process whereby decreasing mobility induces reductions in physical activity. Our study confirms the existing literature linking obesity and diabetes to walking performances, the plausible underlying mechanisms including the excess risk of osteoarthritis in the legs^44^ and lower extremity function in people aged 45-90 with obesity and diabetes.^45^

### Meaning of the study

Our findings suggest that policies to deal with poor socioeconomic circumstances, in addition to common non-communicable disease risk factors, might be critical to strategies for the promotion of healthy aging. As such, this study supports the hypothesis that a successful implementation of the WHO Global Action Plan for the Prevention and Control of Non-Communicable Diseases and policies to reduce socioeconomic adversity might benefit physical functioning of the population. As with standard non-communicable diseases risk factors, the social environment is modifiable by policies at the local, national, and international levels.^46^ ^47^ Examples of potential interventions include promoting early childhood development, tackling poverty and living circumstances, ensuring that all children have access to high quality education, and creating safe school and work environments by legislation.^48^ ^49^ Given that the present study is based on observational data, our study informs about associations but cannot provide evidence of causality. Further research is needed to determine whether interventions targeting non-communicable disease risk factors and adverse socioeconomic circumstances early in life can potentially slow functional decline.

### Conclusions and implications for future research

Current global health policies are targeted towards established risk factors of health, such as smoking and physical inactivity. Much of the evidence of the benefits of reducing these factors involve hard endpoints, such as mortality and morbidity, whereas few studies have focused on additional intervention targets such as socioeconomic circumstances, or broader measures of wellbeing, such as physical functioning. Our findings from cohort studies in Europe, the United States, Latin America, Africa, and Asia address this limitation and show comparable associations of adverse socioeconomic circumstances and standard non-communicable disease risk factors with reduced walking speed, a measure of physical functioning from early old age onwards. Years of functioning lost were greater than years of life lost due to these risk factors. This evidence calls for interventional research on potential benefits of broader health policies dealing with socioeconomic adversity, in addition to standard risk factors.

**What is already known on this topic**

Years of life lost due to unfavourable socioeconomic circumstances and non-communicable disease risk factors have been estimated, but the extent to which such factors affect physical functioning is unknown
In addition to prevention of age related morbidity and premature mortality, minimising the time that people spend living with disability and dependence is a major public health challenge
Walking speed, an indicator of physical functioning, declines with age and is a predictor of survival, hospital admission, and cognitive decline

**What this study adds**

The independent association between socioeconomic status and walking speed in old age is comparable in strength and consistency (across sex and age groups) to those for leading non-communicable disease risk factors
Overall, 4 to 7 years of good physical functioning are lost due to poor socioeconomic circumstances at age 60 and the corresponding loss for other risk factors are 0.5 to 8 years
Physical functioning continues to decline as a function of socioeconomic status and unfavourable risk factors at least until age 85

## Web extra

Extra material supplied by authors

Supplementary information: descriptions of included cohort studies, tables S1-S7, figures S1-S8, and sensitivity analyses using a two step approach

## Notes

Contributors: SS and CC contributed equally to the study. MKi and PV are joint last authors. MK, SS, and PV conceived the study. SS wrote the first and successive drafts of the manuscript. CC modelled and analysed the data. CC, MA, and MJ contributed to study conception and design. SS, MJ, CM, MG, and AIR contributed to data analysis. CC, MJ, MB, HB, GC, MG, GGG, RAK, MGM, AS, MJS, MZ, PV, and MK collected the data. All authors revised the manuscript for important intellectual content. SS and CC had full access to the data and take responsibility for the integrity of the data and the accuracy of the data analysis. SS is the guarantor.

Ethical approval: Not required.

Data sharing: No additional data available.

Transparency: The lead authors (SS and CC) affirm that his manuscript is an honest, accurate, and transparent account of the study being reported; that no important aspects of the study have been omitted; and that any discrepancies from the study as planned (and, if relevant, registered) have been explained.
