# Supplementary material for: Socioeconomic status, non-communicable disease risk factors, and walking speed in older adults: multi-cohort population based study
Source: BMJ. 2018 Mar 23;360:k1046. doi: 10.1136/bmj.k1046 (PMC5865179; doi:10.1136/bmj.k1046)
Supplement: Supplementary file 1 — Supplementary information: descriptions of included cohort studies, tables S1-S7, figures S1-S8, and sensitivity analyses using a two step approach [file strs041537.ww1.pdf]

Appendix 1. Details on the study design and participants of the cohorts included in the analysis

GAZEL. The GAZEL study was established in 1989 among employees of the French national gas and electricity company, Electricité de France-Gaz de France (EDF-GDF). At baseline (1989), 20,625 employees (15,011 men and 5,614 women), aged 35–50, gave consent to participate. The study design consists of an annual questionnaire used to collect data on health, lifestyle, individual, familial, social and occupational factors, and life events. http://www.gazel.inserm.fr/en/

CONSTANCES. The CONSTANCES cohort was established in late 2012. Supported by the French National Research Agency (ANR-11-INBS-0002), it was designed as a randomly selected representative sample of French adults aged 18-69 years at inception; 200,000 subjects will be included over a five-year period. At enrolment, the participants fill questionnaires collecting data on health, lifestyle, individual, familial, social and occupational factors, and life events and benefits from a comprehensive health examination. The follow-up includes a yearly self-administered questionnaire, a health examination every 5 years and an annual linkage to social and health national databases (Zins et al, 2015). http://www.constances.fr/index_EN.php

WHITEHALL II. The Whitehall II study was established in 1985 to examine the socioeconomic gradient in health among 10,308 London-based civil servants (6,895 men and 3,413 women) aged 35–55. Baseline examination (phase 1) took place during 1985–1988, and involved a clinical examination and a self-administered questionnaire containing sections on demographic characteristics, health, lifestyle factors, work characteristics, social support, and life events. https://www.ucl.ac.uk/whitehallII

ELSA. The English Longitudinal Study of Ageing (ELSA) is a panel study of a representative cohort of men and women living in England aged ≥50 years. It was designed as a sister study to the Health and Retirement Study in the USA and is multidisciplinary in orientation, involving the collection of economic, social, psychological, cognitive, health, biological and genetic data. The study commenced in 2002, and the sample has been followed up every 2 years. Data are collected using computer-assisted personal interviews and self-completion questionnaires, with additional nurse visits for the assessment of biomarkers every 4 years. The original sample consisted of 11,391 members ranging in age from 50 to 100 years. http://www.elsa-project.ac.uk/

NHANES. The National Health and Nutrition Examination Survey (NHANES) is a program of studies designed to assess the health and nutritional status of adults and children in the United States. The survey is unique in that it combines interviews and physical examinations. NHANES is a major program of the National Center for Health Statistics (NCHS). NCHS is part of the Centers for Disease Control and Prevention (CDC) and has the responsibility for producing vital and health statistics for the Nation.The NHANES program began in the early 1960s and has been conducted as a series of surveys focusing on different population groups or health topics. In 1999, the survey became a continuous program that has a changing focus on a variety of health and nutrition measurements to meet emerging needs. The survey examines a nationally representative sample of about 5,000 persons each year. These persons are located in counties across the country, 15 of which are visited each year. The NHANES interview includes demographic, socioeconomic, dietary, and health-related questions. The examination component consists of medical, dental, and physiological measurements, as well as laboratory tests administered by highly trained medical personnel. http://www.cdc.gov/nchs/nhanes/

HRS. The University of Michigan Health and Retirement Study (HRS) is a longitudinal panel study that surveys a representative sample of approximately 20,000 people in America over the age of 50

every two years. Supported by the National Institute on Aging (NIA U01AG009740) and the Social Security Administration, the HRS explores the changes in labor force participation and the health transitions that individuals undergo toward the end of their work lives and in the years that follow. Since its launch in 1992, the study has collected information about income, work, assets, pension plans, health insurance, disability, physical health and functioning, cognitive functioning, and health care expenditures. Through its unique and in-depth interviews, the HRS provides an invaluable and growing body of multidisciplinary data that researchers can use to address important questions about the challenges and opportunities of aging. http://hrsonline.isr.umich.edu/index.php?p=start

MIDUS. The first national survey of Midlife Development in the U.S. (MIDUS) was conducted in 1995/96 by the MacArthur Foundation Research Network on Successful Midlife Development. The study was conceived by a multidisciplinary team of scholars from fields of psychology, sociology, epidemiology, demography, anthropology, medicine, and health care policy. Their collective aim was to investigate the role of behavioral, psychological, and social factors in accounting for age-related variations in health and well-being in a national sample of Americans. In addition to a national probability sample (N = 3,487), the study included over-samples in select metropolitan areas (N = 757), a sample of siblings (N = 950) of the main respondents, and a national sample of twin pairs (N=1,914). http://midus.wisc.edu/scopeofstudy.php

WLSG and **WLSS**. The Wisconsin Longitudinal Study has followed a random sample of 10,317 participants (5,326 women, 4,991 men) who were born between 1937 and 1940 and who graduated from Wisconsin high schools in 1957 (graduate sample, WLSG). After baseline data collection in 1957, survey data have been collected from the participants or their parents in 1964, 1975, 1992/3, and 2003/5. The present study used data from the 1993/1994 follow-up. The WLS sample is broadly representative of white, non-Hispanic American men and women who have completed at least a high school education (among Americans aged 50 to 54 in 1990 and 1991, approximately 66 percent were non-Hispanic white persons who completed at least 12 years of schooling). It is estimated that about 75 percent of Wisconsin youth graduated from high school in the late 1950s – everyone in the primary WLS sample graduated from high school. In addition to the main sample of the 1957 high school graduates, the WLS has also collected data on a selected sibling of a sample of the graduates (sibling sample, WLSS). The data collection in adulthood has been very similar although not entirely identical for the siblings as for the graduates. For the present purposes, the sibling sample was analyzed separately from the graduate sample, because the sampling frame of the individuals for the graduate cohort and sibling cohort was considered to sufficiently to justify the decision of not combining the samples. Baseline data were from the 1994/1995 follow-up.

CRELES. The Costa Rican Longevity and Healthy Aging Study (CRELES, or "Costa Rica Estudio de Longevidad y Envejecimiento Saludable") is a set of nationally representative longitudinal surveys of health and lifecourse experiences of older Costa Ricans. CRELES is part of the growing set of Health and Retirement Surveys being conducted around the world. Costa Rica is of particular interest to study given its high longevity: life expectancy is greater than that of the United States, despite being a middle income country with about one-fifth the per capita income and one-tenth the per capita health spending. CRELES is now composed of multiple waves of data from two birth cohorts. The original CRELES Pre-1945 cohort is a nationally representative sample of nearly 3,000 Costa Rican residents born in 1945 or before. Baseline CRELES household interviews were conducted primarily in 2005, with 2-year follow-up interviews in 2007 and 2009. The study was conducted by the University of Costa Rica's Centro Centroamericano de Población (CCP) in collaboration with the Instituto de Investigaciones en Salud (INISA), with the support of the Wellcome Trust (grant 072406). The CRELES 1945-1955 Retirement Cohort (RC) is a sample of Costa Rican residents born 1945-1955, first interviewed starting in 2010 with a second wave starting in 2012. The sample

includes about 2,800 baseline long-form interviews with targeted age-eligibles plus about 1,400 interviews with their spouses (regardless of age), conducted between January 2010 and December 2011. The CRELES-RC also includes a supplemental sample of short-form interviews conducted between January 2012 and January 2013 with about 500 initially non-responding target individuals so as to study response-rate patterns which may be especially systematic in working age populations. This study was again conducted by the University of Costa Rica's Centro Centroamericano de Población, in collaboration with the University of California at Berkeley, with funding from the U.S. National Institute on Aging. http://www.creles.berkeley.edu/

HAALSI. The Health and Aging in Africa: longitudinal studies of INDEPTH communities study is based in the Agincourt HDSS site, a sub-district of rural Mpumalanga Province comprising some 116,000 people living in 19,000 households and 31 villages in an area of ~450 km2. HAALSI investigates drivers and consequences of noncommunicable diseases and HIV among older adults. The study setting, Agincourt subdistrict of rural northeast South Africa, is an area of poverty, low education, high unemployment, and consequent labor migration. The population of 110,000 has been followed since 1992 under the Agincourt Health and Socio-Demographic Surveillance System. A total of 5,059 women and men aged 40 and older were enrolled in HAALSI between November 2014 and December 2015. The survey response rate was 85.9%. Respondents completed a household questionnaire capturing demographics, education, employment history, cognitive and physical functioning, self-reported health history, and social, HIV, and cardiometabolic risk factors. https://haalsi.org/

HEPESE. The Hispanic Established Populations for the Epidemiologic Study of the Elderly comprises the fourth follow-up of the baseline Hispanic EPESE, 1993-1994: [ARIZONA, CALIFORNIA, COLORADO, NEW MEXICO, AND TEXAS]. The baseline Hispanic EPESE collected data on a representative sample of community-dwelling Mexican-Americans, aged 65 years and older, residing in the five south-western states of Arizona, California, Colorado, New Mexico, and Texas. The primary purpose of the series was to provide estimates of the prevalence of key physical health conditions, mental health conditions, and functional impairments in older Mexican Americans and to compare these estimates with those for other populations. The Hispanic EPESE provides data on risk factors for mortality and morbidity in Mexican Americans in order to contrast how these factors operate differently in non-Hispanic White Americans, African Americans, and other major ethnic groups. The public-use data cover demographic characteristics (age, sex, type of Hispanic race, income, education, marital status, number of children, employment, and religion), height, weight, social and physical functioning, chronic conditions, related health problems, health habits, self-reported use of dental, hospital, and nursing home services, and depression. Subsequent follow-ups provide a cross-sectional examination of the predictors of mortality, changes in health outcomes, and institutionalization, and other changes in living arrangements, as well as changes in life situations and quality of life issues. During this 5th Wave, 2004-2005, reinterviews were conducted either in person or by proxy, with 1,167 of the original respondents. This 4th follow-up includes an additional sample of 902 Mexican Americans aged 75 and over with higher average-levels of education than those of the surviving cohort, increasing the total number of respondents to 2,069. By diversifying the cohort of those aged 75 and older, a better understanding can be gained of the influence of socioeconomic and cultural variations on the lives and health of older Mexican Americans.

SAGE. WHO's Study on global AGEing and adult health is a longitudinal study with nationally representative samples of persons aged 50+ years in China, Ghana, India, Mexico, Russia and South Africa, with a smaller sample of adults aged 18-49 years in each country for comparisons. Instruments are compatible with other large high-income country longitudinal ageing studies. Face-to-face interviews conducted in China (2008–10), Ghana (2008–09), India (2007–08), Mexico (2009–10), the Russian Federation (2007–10) and South Africa (2007–08). Half the interviews in China were

completed using a computer-assisted personal interview (CAPI) and the other half using paper and pencil. SAGE Mexico used CAPI throughout and the other four countries used paper and pencil format for all interviews. Multistage cluster sampling strategies were used in all countries where, except for Mexico, households were classified into one of two mutually exclusive categories: (1) all persons aged 50 years and older were selected from households classified as '50+ households'; and (2) one person aged 18–49 years was selected from a household classified as an '18–49 household'. The arrangement in Mexico was similar, but included supplementary and replacement samples to account for losses to follow up in selected sampling units since Wave 0 (more sampling details provided when accessing the data through http://apps.who.int/healthinfo/systems/surveydata/index.php/catalog). The sample in India is also representative at the sub-national and sub-state levels for the selected states. Household enumerations were carried out for the final sampling units. One household questionnaire was completed per household—where a household informant and individual respondent need not be the same individual. One individual was selected from 18–49 households, whereas for 50+ households all individuals aged 50+ were invited to complete the individual interview. Proxy respondents were identified for selected individuals who were unable to complete the interview. Household-level analysis weights and person-level analysis weights were calculated for each country, which included sample selection and a post-stratification factor. Post-stratification correction techniques used the most recent population estimates provided by the national statistical offices. The pooled Wave 1 six-country totals for individual respondents included 34,124 respondents aged 50+ and 8,340 aged 18–49.

SEBAS. The Social Environment and Biomarkers of Aging Study (SEBAS) in Taiwan, 2000 and 2006, provides information regarding the health and well-being of older persons in Taiwan. Taiwan has undergone rapid demographic, social, and economic changes, becoming a highly urbanized and industrial society with a growing population of persons age 65 or older. SEBAS explores the relationship between life challenges and mental and physical health, the impact of social environment on the health and well-being of the elderly, as well as biological markers of health and stress. The study collected self-reports of physical, psychological, and social well-being, plus extensive clinical data based on medical examinations and laboratory analyses. Examination of health outcomes included chronic illnesses, functional status, psychological well-being, and cognitive function. Questions regarding life challenges focused on perceived stress, economic difficulties, security and safety, and the consequences of a major earthquake. Biological markers were used to identify cardiovascular risk factors, metabolic process measures, immune-system activity, the hypothalamic-pituitary adrenal axis, and sympathetic nervous system activity. Two rounds of biomarker data collected in 2000 and 2006 were complemented by face-to-face interviews with the participants. Demographic and background variables included age, sex, education, ethnicity, occupation, and residency. Additional information about the Social Environment and Biomarkers of Aging Study can be found at the Georgetown University Center for Populations and Health Web site.

SHARE. The Survey of Health, Ageing and Retirement in Europe (SHARE) is a multidisciplinary and cross-national panel database of micro data on health, socio-economic status and social and family networks of approximately 123,000 individuals aged 50 or older (more than 293,000 interviews). SHARE covers 27 European countries and Israel. To date, SHARE has collected more than 293,000 interviews in four panel waves on current living circumstances and one wave on retrospective life histories (SHARELIFE). The first wave was collected in 2004/2005, the second in 2006/2007, SHARELIFE in 2008/2009, the fourth wave mainly in 2011 and the fifth wave in 2013. Up to the present 20 countries participated in SHARE. However, not all countries were part of each wave and also the timing of data collection differs between countries. Table S1 gives information on the participation of countries and time of data collection for Wave 1 to Wave 5.

NSHAP. The National Social Life, Health, and Aging Project (NSHAP) is a longitudinal, population-based study of health and social factors, aiming to understand the well-being of older, community-dwelling Americans by examining the interactions among physical health and illness, medication use, cognitive function, emotional health, sensory function, health behaviors, social connectedness, sexuality, and relationship quality. NSHAP provides policy makers, health providers, and individuals with useful information and insights into these factors, particularly on social and intimate relationships. The study contributes to finding new ways to improve health as people age. To date there are two waves of NSHAP data available to researchers, and funding has now been awarded for a third wave of data collection. In 2005 and 2006, NORC and Principal Investigators at the University of Chicago conducted the first wave of NSHAP, completing more than 3,000 interviews with a nationally representative sample of adults born between 1920 and 1947 (aged 57 to 85 at the time of Wave 1 interview). In 2010 and 2011, nearly 3,400 interviews were completed for Wave 2 with these Wave 1 Respondents, Wave 1 Non-Interviewed Respondents, and their spouses or cohabiting romantic partners. The second wave of NSHAP is essential to understanding how social and biological characteristics change. By eliciting a variety of information from respondents over time, NSHAP provides data that will allow researchers in a number of fields to examine how specific factors may or may not affect each other across the life course.

TILDA. The Irish Longitudinal Study on Ageing (TILDA) is a large prospective cohort study examining the social, economic, and health circumstances of 8,175 community-dwelling adults aged 50 years and older resident in the Republic of Ireland. The sample was generated using a three-stage selection process and the Irish Geodirectory as the sampling frame. The Irish Geodirectory is a comprehensive listing of all addresses in the Republic of Ireland which is compiled by the national post service and ordnance survey Ireland. Subdivisions of district electoral divisions pre-stratified by SES, age, and geographical location served as the primary sampling units. The second stage involved the selection of a random sample of 40 addresses from within each PSU resulting in an initial sample of 25,600 addresses. The third stage involved the recruitment of all members of the household aged 50 years and over. Consequently, the response rate was defined as the proportion of households including an eligible participant from whom an interview was successfully obtained. A response rate of 62.0% was achieved at the household level. There were three components to the survey. Respondents completed a computer-assisted personal interview (CAPI) and a separate self-completion paper and pencil module which collected information that was considered sensitive. All participants were invited to undergo a separate health assessment at one of two national centers using trained nursing staff. At the initial interview, respondents were invited to undergo a detailed health assessment at one of two national centers in Dublin and Cork using trained nursing staff. If a respondent could not attend the health center but was agreeable to completing a health assessment, a trained nurse administered a subset of the tests in the respondent's home. The Trinity College Dublin Research Ethics Committee granted ethical approval for the study.

EPIPORTO. The EPIPorto study was initiated in 1999 and recruited 2,485 adult dwellers aged 18 years or more in the city of Porto, northwest of Portugal. Briefly, simple random digit dialing of landline telephones was used to select households. The vast majority of houses (>95%) had a landline telephone at the time of this procedure. We used a table of random numbers to define the last four digits that are specific to individual houses, assuming the local prefix codes to limit the universe to the city of Porto. Non-existing numbers, those corresponding to fax numbers or telephone numbers of non-individual subscribers were ignored. The household was considered unreachable after at least four dialing attempts at different hours and including week and weekend days. Within each household, a permanent resident aged 18 years or more was selected using simple random sampling. The proportion of participation was 70%. A follow-up evaluation was conducted from 2005 to 2008

(participation rate=68% of the baseline sample), by trained interviewers, using structured questionnaires and forms, following the same protocol for data collection as at baseline. In both evaluations, participants were invited to visit our Department at Medical School for an interview, which included a questionnaire on social, demographic, behavioural and clinical data, and a physical examination including blood collection.

Appendix 2. Details on measures

Smoking was self-reported and was categorized into current smoking for individuals currently smoking, former smoking for individuals not currently smoking but who smoke in the past, and never smoking for individuals who never smoke.

Alcohol consumption was measured in alcohol units. For most cohorts, it was the average number of alcohol units over a week. For ELSA, it was the highest intake over a week. High alcohol intake was defined as a consumption exceeding 21 alcohol units for men and 14 for women. Abstinence was defined as no alcohol consumption, and moderate intake was defined as consumption less or equal 21 alcohol units for men and 14 for women. For CRELES Pre 1945 and CRELES-RC, the strata were determined based on drinking frequency only. In SHARE, heavy alcohol consumption was assessed based on frequency of drinking more than 2 units of beer, wine, and liquor.

Physical inactivity was considered as none/light activity only for ELSA, as less than 1 hour of moderate and vigorous physical activity per week in WHITEHALL II and TILDA, as not practicing sport in GAZEL, EPIPORTO and CRELES Pre 1945. Physical inactivity was determined as no moderate or vigorous physical activity in CRELES-RS, HAALSI, NHANES, and SHARE; no moderate or vigorous physical activity at least once a week in the HRS, MIDUS, WLSG, and WLSS; no moderate or vigorous physical activity and no walking or biking more than 10 minutes continuously in SAGE; no regular exercise in SEBAS; no or less than once a month vigorous physical activity in NSHAP.

Body mass index (BMI) was measured as weight (Kg) divided by the square of height (m^2). Obesity is defined as BMI$\geq$30; Overweight is defined as BMI$\geq$25 and BMI<30; Normal BMI is defined as BMI$\geq$ 18.5 and BMI<25. For GAZEL, HRS, MIDUS, SHARE, WLSG and WLSS the values were self-reported, measured in all other ones. Hypertension was defined as the presence of at least one of the following conditions: systolic blood pressure$\geq$140 mmHg, recorded diastolic blood pressure$\geq$90 mmHg, current intake of anti-hypertensive medication, self-report. For GAZEL and CRELES-RC only the self-reported information was available. Blood pressure was self-reported in WLSG, and WLSS, and measured as the mean of two or three readings in the remaining studies. Knowledge of anti-hypertensive treatment was available in HAALSI, CRELES Pre 1945, TILDA, HRS, MIDUS, NHANES1999, NHANES2001, SAGE, and SHARE.

Diabetes was defined as the presence of at least one of the following conditions: fasting glucose$\geq$7 mmol/L, 2h postload glucose$\geq$11.1 mmol/L, glycated hemoglobin $\geq$6.5%, self-report. All had self-reported information, WHITEHALL-II and HAALSI further has both fasting glucose and 2h post load glucose, ELSA and CRELES-RC have glycated hemoglobin, CRELES Pre 1945 fasting glucose and glycated haemoglobin. In the NHANES, diabetes was determined based on fasting glucose and glycated haemoglobin or taking medication. In SEBAS, diabetes was determined based on glycated haemoglobin or taking medication.

Ethnicity was measured as white, Asian, black African or other. Out of 68,112 participants with known ethnicity, 52.9% were white, 22.9% Asian and 16.7% black African.

Self-rated health status was categorized as good or not good. Out of 104,627 participants with known health status, 70.3% stated to have good health and were 2.7 years younger than those with poor health. There were no differences between men and women. Most studies had a question about health status based on five levels, but HEPESE with four levels. We considered as good health those participants stating to have good or very good or excellent health, and not good otherwise (fair, poor or very poor).

Appendix 3. Supplementary analyses

Table S1. Characteristics of the walking speed test. Reasons for not completing the test due to health included inability to walk alone, recent surgery, injury, being on a wheelchair, or other. Other reasons for not completing the test included equipment problems, not suitable space available to perform the test, interruption, or other. Outliers were defined as normal walking speed >5m/s (N=48 in CONSTANCES and N=1 in GAZEL).

Study	wave	Test setup	Test participants N men/women	Test not completed for health reasons N men/women	Test not completed for refusal N men/women	Test not completed for other reasons N men/women	Missing value of walking speed - unknown or outlier N men/women
GAZEL	Wave 21	Start standing, after walking 1m the time of walking next 3m timed via photoelectric devices. 1 trial	2,164 / 693	0/1	1/0	1/2	4/1
ELSA	Wave 2	Start standing with both feet together, time of walking next 2.44m timed using a stopwatch. 2 trials	2,577/3,206	46/57	36/30	14/24	37/59
WHITEHALL II	Wave s7	Start standing with both feet together, time of walking next 2.44m timed with a stopwatch. 3 trials	4,777/2,020	NA	NA	NA	393/209
NHANES III	Baseline	Time of walking 2.44m. 2 trials	2,612/2,791	75/99	0/0	60/78	0/0
NHANES 1999	Baseline	Time of walking 6.1m timed using a stopwatch. 1 trial	1,060/1,090	23/38	11/13	60/70	0/1
NHANES 2001	Baseline	Time of walking 6.1m timed using a stopwatch. 1 trial	1,146/1,147	21/33	15/11	95/91	0/1
HRS	Waves K and L	Time of walking 6.1m timed using a stopwatch. 2 trials	3,828/5,246	55/113	77/115	301/456	178/115
MIDUS	Biomarker project and wave 2	Start standing with both feet together, time of walking 15.24m measured with a stopwatch. 2 trials	477/577	0/2	0/0	1/1	1/2
WLSG	Year 2011	Time of walking 2.5m. 2 trials	2,697/3,077	NA	NA	NA	64/71
WLSS	Year 2011	Time of walking 2.5m. 2 trials	1,572/1,684	NA	NA	NA	39/39
CONSTANCES	Baseline	Start standing, after walking 1m the time of walking next 3m measured via photoelectric devices. 1 trial	16,040/17,631	14/11	0/1	118/154	25/24
CRELES-RC	Baseline	Time of rising from a chair and walking 3m. 1 trial	1,716/2,421	0/0	5/6	8/8	128/148
CRELES Pre 1945	Baseline	Time of rising from a chair and walking 3m. 1 trial	1,293/1,534	0/0	0/1	7/14	237/334

HAALSI	Baseline	Time of walking 2.5m. 1 trial	2,345/2,714	63/81	75/78	0/0	31/37
HEPESE	Baseline	Start standing, time of walking 2.44m, timed with stopwatch. 1 trial	1,201/1,687	0/0	6/12	125/212	39/59
SEBAS	Wave 2	Time of walking 3m, timed with stopwatch. 2 trials	679/605	11/4	5/13	31/32	0/0
NSHAP	Wave 2	Time of rising from a chair and walking 3m and back, timed as 2 trials.	1,538/1,839	19/22	48/66	18/34	0/0
SAGE China	Baseline	Time of walking 4m. 1 trial	6,702/7,344	34/23	78/104	0/0	5/6
SAGE Ghana	Baseline	Time of walking 4m. 1 trial	2,678/2,323	40/39	17/15	0/0	53/58
SAGE India	Baseline	Time of walking 4m. 1 trial	4,286/6,760	0/0	0/0	0/0	96/131
SAGE Mexico	Baseline	Time of walking 4m. 1 trial	978/1,613	67/110	27/24	0/0	67/95
SAGE Russia	Baseline	Time of walking 4m. 1 trial	1,525/2,725	12/23	351/631	0/0	8/18
SAGE South Africa	Baseline	Time of walking 4m. 1 trial	1,726/2,322	25/33	39/49	0/0	122/164
SHARE Austria	Baseline	Time of walking 2.5m. 2 trials	110/214	36/109	21/34	6/2	3/2
SHARE Belgium	Baseline	Time of walking 2.5m. 2 trials	332/468	83/140	27/45	11/22	17/8
SHARE Denmark	Baseline	Time of walking 2.5m. 2 trials	145/213	32/55	9/9	1/9	7/4
SHARE France	Baseline	Time of walking 2.5m. 2 trials	268/407	67/97	18/31	14/17	13/12
SHARE Germany	Baseline	Time of walking 2.5m. 2 trials	222/332	86/154	33/34	3/15	5/4
SHARE Greece	Baseline	Time of walking 2.5m. 2 trials	205/368	70/133	25/44	5/30	4/10
SHARE Israel	Baseline	Time of walking 2.5m. 2 trials	244/283	97/117	27/46	12/8	21/29
SHARE Italy	Baseline	Time of walking 2.5m. 2 trials	203/299	59/107	44/61	3/7	7/7
SHARE Netherlands	Baseline	Time of walking 2.5m. 2 trials	226/304	46/106	13/14	7/10	23/17
SHARE Spain	Baseline	Time of walking 2.5m. 2 trials	244/421	56/126	26/43	11/11	4/4
SHARE Sweden	Baseline	Time of walking 2.5m. 2 trials	294/324	86/110	33/27	17/18	15/12
SHARE Switzerland	Baseline	Time of walking 2.5m. 2 trials	75/113	16/22	6/7	1/2	5/8

TILDA	Baseline	Start standing, after walking 2.5m the time of walking next 4.88m measured via embedded pressure sensors. 2 trials	3,780/4,724	0/0	10/12	28/34	1,445/1,784
EPIPORTO	Wave 3	Start standing, after walking 4.5m the time of walking next 7.65m measured via stopwatch. 2 trials	396/599	4/4	0/2	2/12	9/17

Table S2. Years of functioning lost by 60 years of age due to suboptimal risk factors – COMPARISON BETWEEN MODELS ADJUSTING FOR PHYSICAL INACTIVITY (PI) AND NOT ADJUSTING FOR PHYSICAL INACTIVITY

	MEN			WOMEN		
	**PI-adjusted**	**Non adjusted for PI**		**PI-adjusted**	**Non adjusted for PI**	
**Risk Factor**	YFL (95% CI)	YFL (95% CI)	N	YFL (95% CI)	YFL (95% CI)	N
Low SES	-5.5 (-7.6,-4.2)	-5.9 (-8.2,-4.6)	51,159	-3.9 (-5.3,-3.0)	-4.3 (-5.7,-3.3)	49,809
Obesity	-4.3 (-5.9,-3.2)	-4.8 (-6.5,-3.6)	48,671	-6.6 (-8.4,-5.4)	-7.2 (-9.0,-5.9)	47,448
Current smoking	-2.9 (-4.1,-2.1)	-3.3 (-4.6,-2.4)	50,309	-0.5 (-1.2,0.1)	-0.8 (-1.5,-0.2)	48,884
High alcohol intake	-0.4 (-1.3,0.4)	-0.6 (-1.5,0.2)	47,754	0.4 (-0.5,1.4)	0.2 (-0.7,1.3)	46,056
Hypertension	-1.8 (-2.6,-1.2)	-2.0 (-2.9,-1.4)	51,033	-2.5 (-3.4,-1.9)	-2.7 (-3.6,-2.1)	49,687
Diabetes	-4.6 (-6.5,-3.4)	-5.0 (-6.9,-3.7)	50,998	-5.3 (-7.0,-4.1)	-5.7 (-7.5,-4.4)	49,591

Table S3. Years of functioning lost by 60 years of age due to suboptimal risk factors – COMPARISON BETWEEN MODELS ADJUSTING FOR ETHNICITY AND NOT ADJUSTING FOR ETHNICITY

	MEN			WOMEN		
	**Ethnicity-adjusted**	**Non adjusted for ethnicity**		**Ethnicity-adjusted**	**Non adjusted for ethnicity**	
**Risk Factor**	YFL (95% CI)	YFL (95% CI)	N	YFL (95% CI)	YFL (95% CI)	N
Low SES	-9.4 (-13.1,-7.1)	-10.9 (-15.2,-8.3)	35,824	-7.1 (-10.3,-5.1)	-8.9 (-12.5,-6.5)	32,288
Obesity	-7.8 (-11.0,-5.7)	-7.8 (-11.1,-5.6)	32,875	-10.4 (-13.6,-8.1)	-11.6 (-15.0,-9.1)	29,792
Current smoking	-3.2 (-5.2,-2.0)	-3.6 (-5.8,-2.2)	34,857	-2.7 (-4.9,-1.1)	-2.3 (-4.5,-0.5)	31,364
High alcohol intake	-0.8 (-3.1,1.3)	-0.6 (-3.2,1.8)	32,608	2.1 (-1.2,5.9)	2.6 (-1.0, 6.8)	29,107
Hypertension	-3.4 (-5.6,-2.2)	-4.0 (-6.4,-2.5)	35,602	-4.8 (-7.4,-3.3)	-5.9 (-8.8,-4.1)	32,016
Diabetes	-6.9 (-10.1,-4.7)	-7.9 (-11.4,-5.5)	35,560	-8.7 (-12.1,-6.4)	-10.3 (-14.1,-7.8)	31,920
Physical inactivity	-8.4 (-11.5,-6.4)	-9.2 (-12.8,-7.0)	33,122	-8.4 (-11.8,-6.3)	-9.6 (-13.6,-7.2)	29,635

Table S4. Years of functioning lost by 60 years of age due to suboptimal risk factors – COMPARISON BETWEEN MODELS ADJUSTING FOR HEALTH STATUS AND NOT ADJUSTING FOR HEALTH STATUS

	MEN			WOMEN		
	**Health status-adjusted**	**Non adjusted for health status**		**Health status-adjusted**	**Non adjusted for health status**	
**Risk Factor**	YFL (95% CI)	YFL (95% CI)	N	YFL (95% CI)	YFL (95% CI)	N
Low SES	-5.5 (-7.8,-4.2)	-6.2 (-8.8,-4.7)	52,893	-3.9 (-5.2,-3.0)	-4.6 (-6.2,-3.6)	51,734
Obesity	-4.6 (-6.3,-3.5)	-5.0 (-6.8,-3.8)	50,348	-6.9 (-8.7,-5.7)	-7.6 (-9.7,-6.1)	49,280
Current smoking	-2.5 (-3.6,-1.8)	-3.0 (-4.2,-2.1)	51,697	-0.3 (-1.0,0.4)	-0.6 (-1.4,0.0)	50,415
High alcohol intake	-0.6 (-1.5,0.3)	-0.6 (-1.5,0.3)	48,854	0.1 (-0.9,1.1)	0.1 (-0.9,1.1)	47,210
Hypertension	-1.9 (-2.7,-1.3)	-2.1 (-3.0,-1.5)	52,683	-2.5 (-3.4,-1.9)	-2.9 (-4.0,-2.3)	51,481
Diabetes	-4.1 (-5.8,-2.9)	-5.1 (-7.2,-3.7)	52,640	-4.8 (-6.4,-3.6)	-6.1 (-8.2,-4.7)	51,381
Physical inactivity	-5.1 (-7.1,-3.9)	-5.8 (-8.1,-4.4)	50,132	-4.8 (-6.3,-3.8)	-5.6 (-7.5,-4.4)	48,880

Table S5. Years of functioning lost by 60 years of age due to suboptimal risk factors – MAXIMUM NUMBER OF PARTICIPANTS FOR EACH RISK FACTOR

	MEN		WOMEN	
**Risk Factor**	YFL (95% CI)	N	YFL (95% CI)	N
Low SES	-6.6 (-9.4,-5.0)	55,255	-4.6 (-6.2,-3.6)	53,852
Obesity	-5.0 (-6.8,-3.9)	57,698	-7.6 (-9.7,-6.3)	61,446
Current smoking	-2.6 (-3.8,-1.9)	57,981	-0.6 (-1.3,0.0)	62,190
High alcohol intake	-0.8 (-1.7,0.0)	55,268	0.0 (-1.0,0.9)	58,824
Hypertension	-2.2 (-3.3,-1.6)	61,152	-2.9 (-3.9,-2.2)	65,496
Diabetes	-5.8 (-8.1,-4.3)	61,074	-6.5 (-8.6,-5.2)	65,379
Physical inactivity	-5.6 (-8.0,-4.4)	56,331	-5.5 (-7.2,-4.4)	60,137

Table S6. Years of functioning lost by 60 years of age due to suboptimal risk factors – MULTIVARIATE MODEL where YFL due to a suboptimal risk factor is adjusted for the number of other risk factors (none, 1, 2, 3, ≥4)

	MEN		WOMEN	
**Risk Factor**	YFL (95% CI)	N	YFL (95% CI)	N
Low SES	-5.3 (-7.2,-4.1)	44,567	-3.8 (-5.1,-2.9)	43,024
Obesity	-3.6 (-5.0,-2.6)	44,567	-6.3 (-8.2,-5.1)	43,024
Current smoking	-2.8 (-4.0,-2.0)	44,567	-0.6 (-1.4,0.1)	43,024
High alcohol intake	0.1 (-0.8,0.9)	44,567	0.5 (-0.6,1.5)	43,024
Hypertension	-1.1 (-1.7,-0.6)	44,567	-1.6 (-2.3,-1.0)	43,024
Diabetes	-3.7 (-5.3,-2.6)	44,567	-4.1 (-5.6,-2.9)	43,024
Physical inactivity	-4.5 (-6.2,-3.4)	44,567	-4.8 (-6.4,-3.7)	43,024

Table S7. Years of functioning lost by 60 years of age due to Obesity – UNDERWEIGHT INCLUDED IN NORMAL BMI SUBPOPULATION

	MEN		WOMEN	
**Risk Factor**	YFL (95% CI)	N	YFL (95% CI)	N
Obesity (with underweight)	-5.0 (-7.0,-3.8)	54,046	-7.4 (-9.5,-6.1)	52,532
Obesity (without underweight)	-5.1 (-7.0,-3.9)	51,939	-7.5 (-9.5,-6.1)	50,628

Figure S1. Years of functioning lost between 60 and 85 years of age due to suboptimal risk factors

The minimally adjusted models were only adjusted for age, age^2, height, year of birth and walked distances; in the mutually adjusted models, SES and the non-communicable diseases risk factors are mutually adjusted.

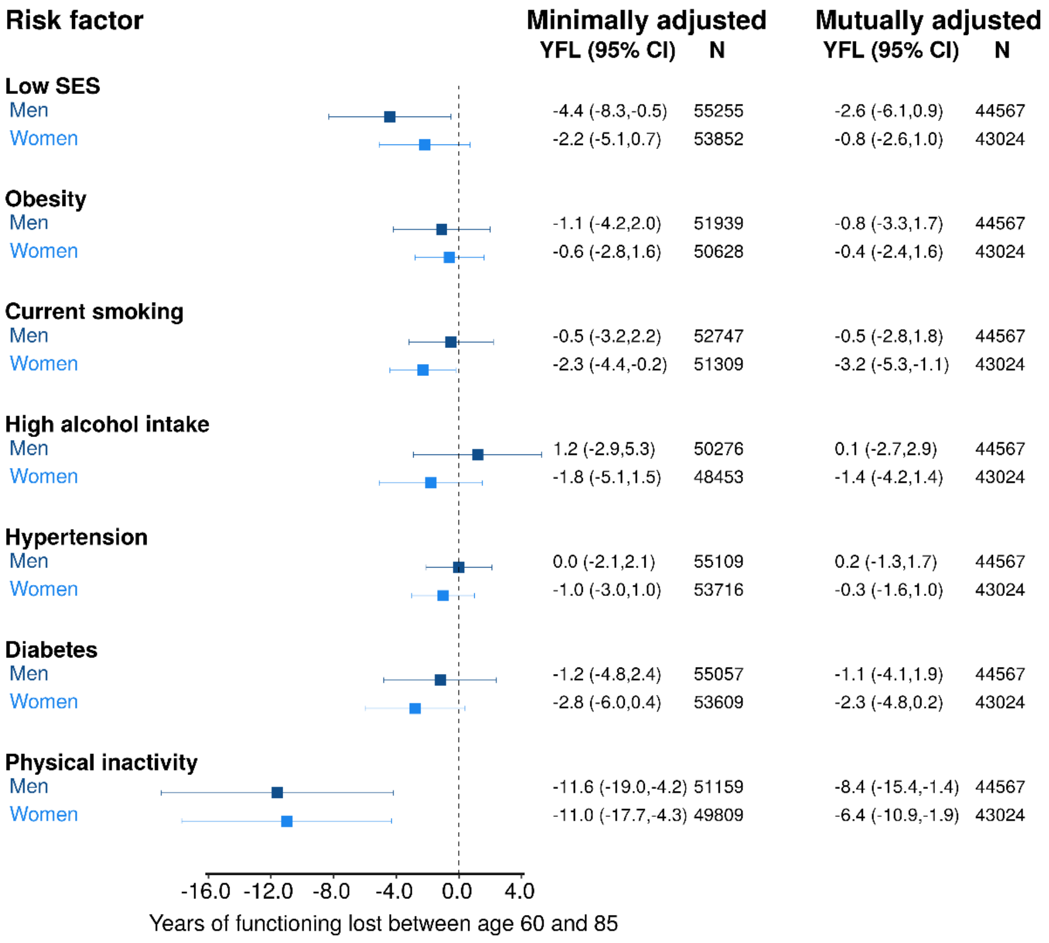

Figure S2. Years of functioning lost by 50 years of age due to suboptimal risk factors

The minimally adjusted models were only adjusted for age, age^2, height, year of birth and walked distances; in the mutually adjusted models, SES and the non-communicable diseases risk factors are mutually adjusted.

Risk factor	Minimally adjusted YFL (95% CI)	Minimally adjusted N	Mutually adjusted YFL (95% CI)	Mutually adjusted N
**Low SES**				
Men	-5.2 (-7.7,-3.7)	55255	-3.8 (-5.6,-2.7)	44567
Women	-3.8 (-5.4,-2.7)	53852	-2.9 (-4.3,-1.8)	43024
**Obesity**				
Men	-4.7 (-6.9,-3.3)	51939	-3.1 (-4.7,-1.8)	44567
Women	-7.2 (-9.5,-5.6)	50628	-5.9 (-8.0,-4.4)	43024
**Current Smoking**				
Men	-2.9 (-4.3,-1.8)	52747	-2.7 (-4.2,-1.7)	44567
Women	0.1 (-0.9,1.2)	51309	0.4 (-0.9,1.7)	43024
**High Alcohol Intake**				
Men	-1.0 (-2.6,0.3)	50276	0.0 (-1.4,1.4)	44567
Women	0.8 (-0.8,2.5)	48453	0.9 (-0.8,2.8)	43024
**Hypertension**				
Men	-2.2 (-3.6,-1.4)	55109	-1.1 (-2.0,-0.3)	44567
Women	-2.6 (-3.8,-1.8)	53716	-0.9 (-1.9,-0.1)	43024
**Diabetes**				
Men	-5.2 (-8.0,-3.4)	55057	-2.9 (-5.0,-1.3)	44567
Women	-5.2 (-7.6,-3.5)	53609	-2.3 (-4.2,-0.6)	43024
**Physical Inactivity**				
Men	-2.8 (-4.2,-1.8)	51159	-1.4 (-2.6,-0.5)	44567
Women	-2.5 (-3.7,-1.7)	49809	-1.7 (-2.9,-0.9)	43024

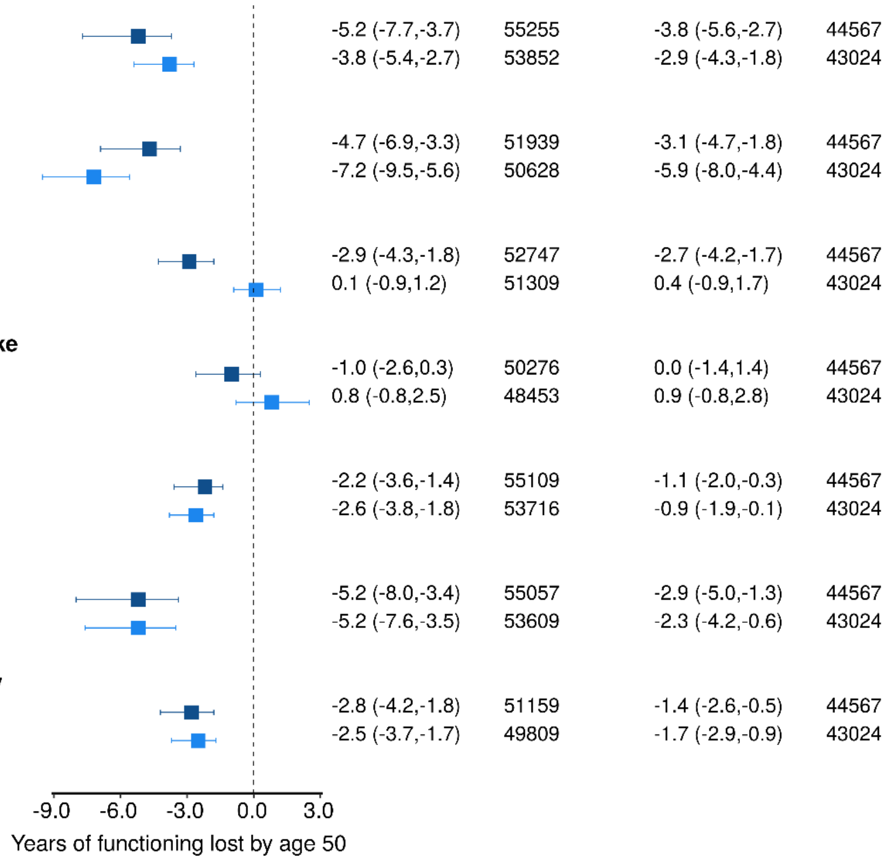

Figure S3. Years of functioning lost by 70 years of age due to suboptimal risk factors

The minimally adjusted models were only adjusted for age, age², height, year of birth and walked distances; in the mutually adjusted models, SES and the non-communicable diseases risk factors are mutually adjusted.

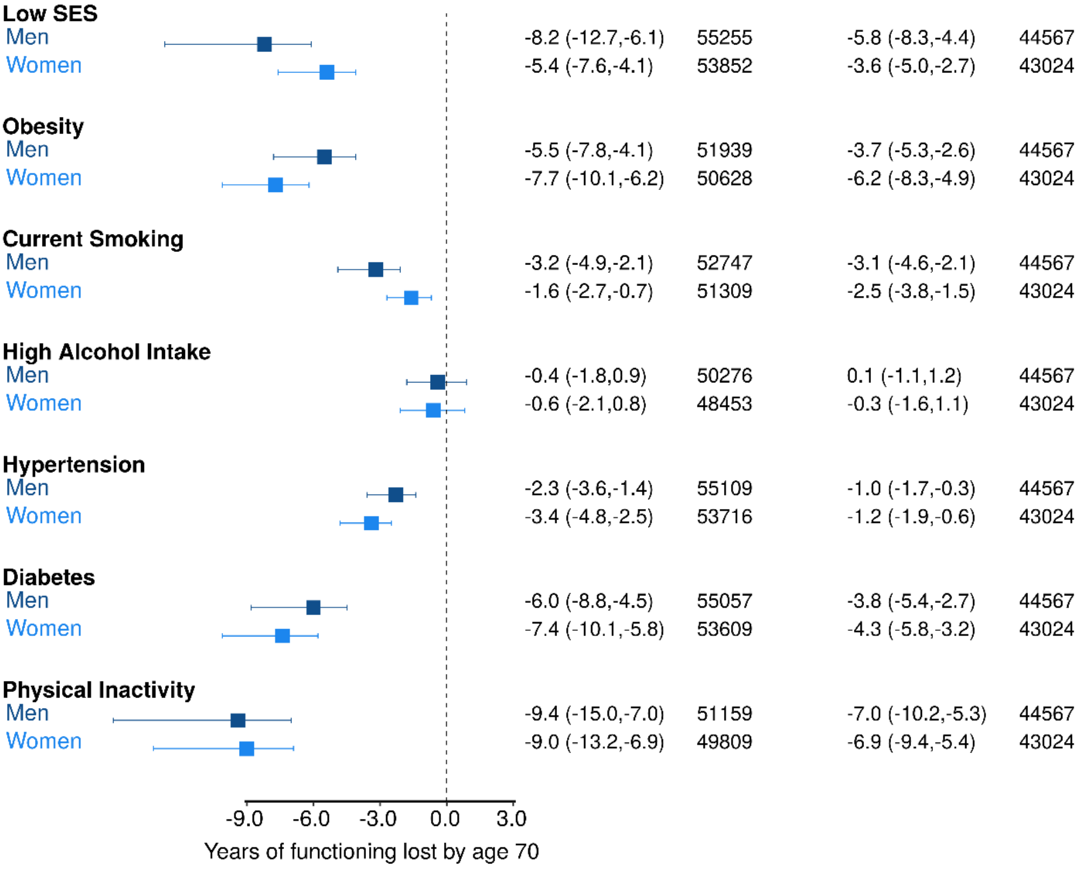

Risk factor	Minimally adjusted YFL (95% CI)	Minimally adjusted N	Mutually adjusted YFL (95% CI)	Mutually adjusted N
**Low SES**				
Men	-8.2 (-12.7,-6.1)	55255	-5.8 (-8.3,-4.4)	44567
Women	-5.4 (-7.6,-4.1)	53852	-3.6 (-5.0,-2.7)	43024
**Obesity**				
Men	-5.5 (-7.8,-4.1)	51939	-3.7 (-5.3,-2.6)	44567
Women	-7.7 (-10.1,-6.2)	50628	-6.2 (-8.3,-4.9)	43024
**Current Smoking**				
Men	-3.2 (-4.9,-2.1)	52747	-3.1 (-4.6,-2.1)	44567
Women	-1.6 (-2.7,-0.7)	51309	-2.5 (-3.8,-1.5)	43024
**High Alcohol Intake**				
Men	-0.4 (-1.8,0.9)	50276	0.1 (-1.1,1.2)	44567
Women	-0.6 (-2.1,0.8)	48453	-0.3 (-1.6,1.1)	43024
**Hypertension**				
Men	-2.3 (-3.6,-1.4)	55109	-1.0 (-1.7,-0.3)	44567
Women	-3.4 (-4.8,-2.5)	53716	-1.2 (-1.9,-0.6)	43024
**Diabetes**				
Men	-6.0 (-8.8,-4.5)	55057	-3.8 (-5.4,-2.7)	44567
Women	-7.4 (-10.1,-5.8)	53609	-4.3 (-5.8,-3.2)	43024
**Physical Inactivity**				
Men	-9.4 (-15.0,-7.0)	51159	-7.0 (-10.2,-5.3)	44567
Women	-9.0 (-13.2,-6.9)	49809	-6.9 (-9.4,-5.4)	43024

Figure S4. Years of functioning lost by 60 years of age due to suboptimal risk factors in high and low/middle income countries for men

The minimally adjusted models were only adjusted for age, age^2, height, year of birth and walked distances; in the mutually adjusted models, SES and the non-communicable diseases risk factors are mutually adjusted.

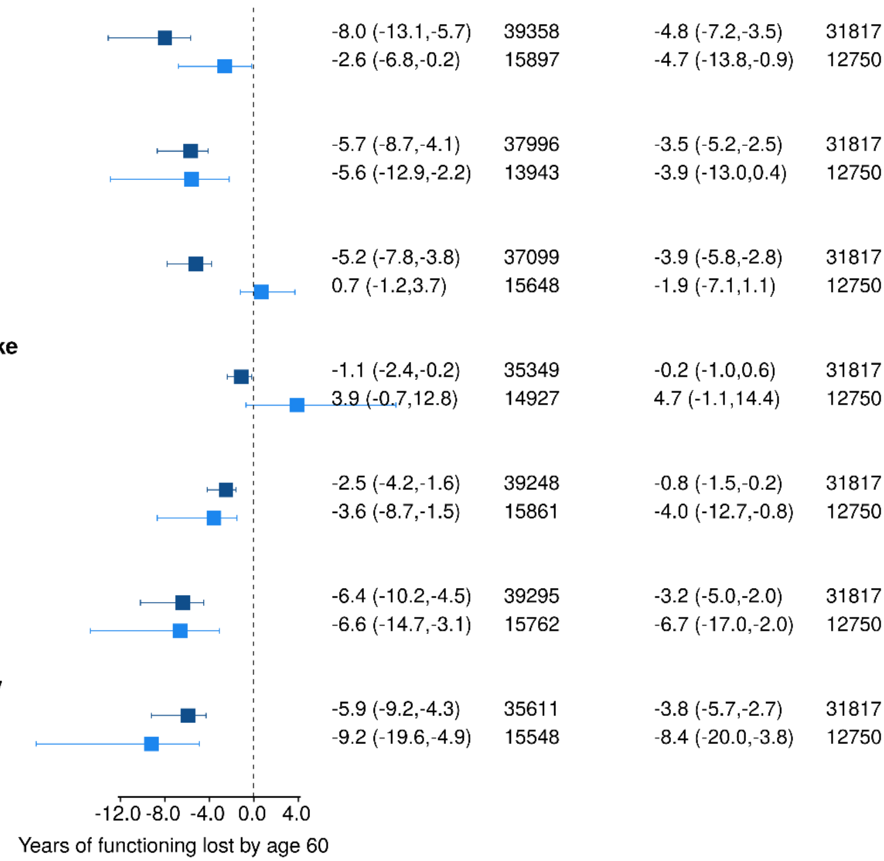

Risk factor	Minimally adjusted YFL (95% CI)	N	Mutually adjusted YFL (95% CI)	N
**Low SES**				
HIC	-8.0 (-13.1,-5.7)	39358	-4.8 (-7.2,-3.5)	31817
LMIC	-2.6 (-6.8,-0.2)	15897	-4.7 (-13.8,-0.9)	12750
**Obesity**				
HIC	-5.7 (-8.7,-4.1)	37996	-3.5 (-5.2,-2.5)	31817
LMIC	-5.6 (-12.9,-2.2)	13943	-3.9 (-13.0,0.4)	12750
**Current Smoking**				
HIC	-5.2 (-7.8,-3.8)	37099	-3.9 (-5.8,-2.8)	31817
LMIC	0.7 (-1.2,3.7)	15648	-1.9 (-7.1,1.1)	12750
**High Alcohol Intake**				
HIC	-1.1 (-2.4,-0.2)	35349	-0.2 (-1.0,0.6)	31817
LMIC	3.9 (-0.7,12.8)	14927	4.7 (-1.1,14.4)	12750
**Hypertension**				
HIC	-2.5 (-4.2,-1.6)	39248	-0.8 (-1.5,-0.2)	31817
LMIC	-3.6 (-8.7,-1.5)	15861	-4.0 (-12.7,-0.8)	12750
**Diabetes**				
HIC	-6.4 (-10.2,-4.5)	39295	-3.2 (-5.0,-2.0)	31817
LMIC	-6.6 (-14.7,-3.1)	15762	-6.7 (-17.0,-2.0)	12750
**Physical Inactivity**				
HIC	-5.9 (-9.2,-4.3)	35611	-3.8 (-5.7,-2.7)	31817
LMIC	-9.2 (-19.6,-4.9)	15548	-8.4 (-20.0,-3.8)	12750

Figure S5. Years of functioning lost by 60 years of age due to suboptimal risk factors in high and low/middle income countries for women

The minimally adjusted models were only adjusted for age, age^2, height, year of birth and walked distances; in the mutually adjusted models, SES and the non-communicable diseases risk factors are mutually adjusted.

Risk factor	Minimally adjusted YFL (95% CI)	Minimally adjusted N	Mutually adjusted YFL (95% CI)	Mutually adjusted N
**Low SES**				
HIC	-5.5 (-8.2,-4.0)	38079	-3.1 (-4.7,-2.2)	29515
LMIC	-2.7 (-5.5,-1.0)	15773	-4.0 (-8.2,-1.9)	13509
**Obesity**				
HIC	-8.4 (-11.5,-6.5)	36185	-6.3 (-9.0,-4.8)	29515
LMIC	-6.7 (-12.0,-4.2)	14443	-5.9 (-11.4,-3.5)	13509
**Current Smoking**				
HIC	-1.4 (-2.5,-0.6)	35852	-1.4 (-2.5,-0.6)	29515
LMIC	2.5 (0.6,5.6)	15457	1.2 (-1.3,4.8)	13509
**High Alcohol Intake**				
HIC	0.0 (-1.0,1.1)	33331	0.3 (-0.7,1.3)	29515
LMIC	0.0 (-8.0,7.8)	15122	0.8 (-9.6,12.4)	13509
**Hypertension**				
HIC	-3.2 (-4.8,-2.3)	37970	-0.8 (-1.6,-0.2)	29515
LMIC	-4.4 (-8.1,-2.6)	15746	-3.0 (-6.5,-1.3)	13509
**Diabetes**				
HIC	-8.5 (-12.4,-6.3)	37979	-4.2 (-6.4,-2.7)	29515
LMIC	-4.7 (-8.5,-2.5)	15630	-3.5 (-7.3,-1.3)	13509
**Physical Inactivity**				
HIC	-6.4 (-9.4,-4.8)	34292	-4.8 (-7.1,-3.5)	29515
LMIC	-4.2 (-7.3,-2.5)	15517	-3.4 (-7.2,-1.8)	13509

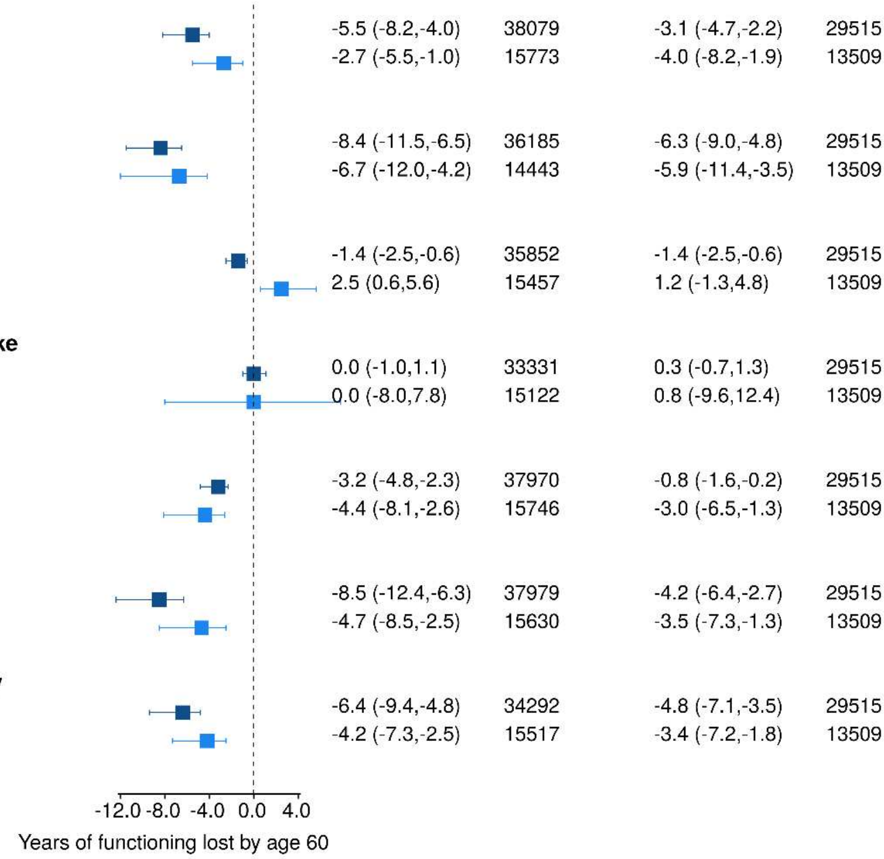

Figure S6. Years of functioning lost by 60 years of age due to suboptimal risk factors in the US

The minimally adjusted models were only adjusted for age, age^2, height, year of birth and walked distances; in the mutually adjusted models, SES and the non-communicable diseases risk factors are mutually adjusted.

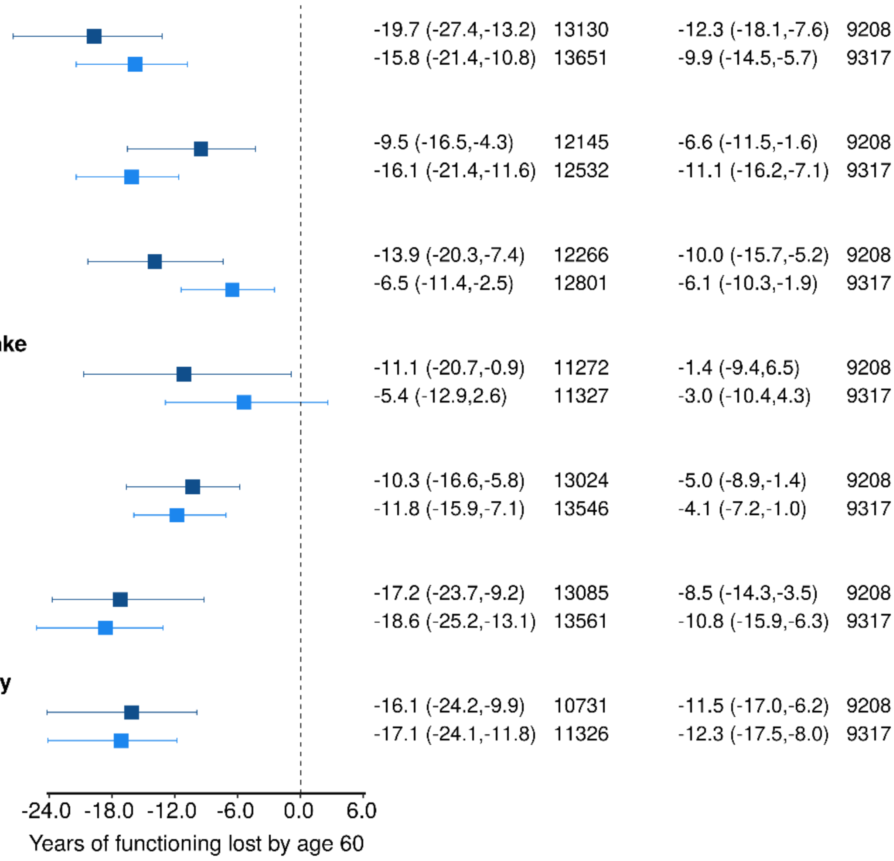

Risk factor	Minimally adjusted YFL (95% CI)	N	Mutually adjusted YFL (95% CI)	N
**Low SES**				
Men	-19.7 (-27.4,-13.2)	13130	-12.3 (-18.1,-7.6)	9208
Women	-15.8 (-21.4,-10.8)	13651	-9.9 (-14.5,-5.7)	9317
**Obesity**				
Men	-9.5 (-16.5,-4.3)	12145	-6.6 (-11.5,-1.6)	9208
Women	-16.1 (-21.4,-11.6)	12532	-11.1 (-16.2,-7.1)	9317
**Current Smoking**				
Men	-13.9 (-20.3,-7.4)	12266	-10.0 (-15.7,-5.2)	9208
Women	-6.5 (-11.4,-2.5)	12801	-6.1 (-10.3,-1.9)	9317
**High Alcohol Intake**				
Men	-11.1 (-20.7,-0.9)	11272	-1.4 (-9.4,6.5)	9208
Women	-5.4 (-12.9,2.6)	11327	-3.0 (-10.4,4.3)	9317
**Hypertension**				
Men	-10.3 (-16.6,-5.8)	13024	-5.0 (-8.9,-1.4)	9208
Women	-11.8 (-15.9,-7.1)	13546	-4.1 (-7.2,-1.0)	9317
**Diabetes**				
Men	-17.2 (-23.7,-9.2)	13085	-8.5 (-14.3,-3.5)	9208
Women	-18.6 (-25.2,-13.1)	13561	-10.8 (-15.9,-6.3)	9317
**Physical Inactivity**				
Men	-16.1 (-24.2,-9.9)	10731	-11.5 (-17.0,-6.2)	9208
Women	-17.1 (-24.1,-11.8)	11326	-12.3 (-17.5,-8.0)	9317

Figure S7. Years of functioning lost by 60 years of age due to suboptimal risk factors in Europe

The minimally adjusted models were only adjusted for age, age^2, height, year of birth and walked distances; in the mutually adjusted models, SES and the non-communicable diseases risk factors are mutually adjusted.

Risk factor	Minimally adjusted YFL (95% CI)	N	Mutually adjusted YFL (95% CI)	N
**Low SES**				
Men	-6.0 (-12.1,-3.8)	25714	-4.3 (-7.8,-2.8)	22609
Women	-3.9 (-7.2,-2.6)	24097	-2.6 (-4.7,-1.6)	20198
**Obesity**				
Men	-5.4 (-10.3,-3.5)	25348	-3.8 (-6.8,-2.4)	22609
Women	-7.1 (-11.6,-5.2)	23330	-6.2 (-10.7,-4.3)	20198
**Current Smoking**				
Men	-4.3 (-7.9,-2.9)	24319	-3.4 (-6.0,-2.1)	22609
Women	-1.0 (-2.2,-0.2)	22720	-1.2 (-2.5,-0.3)	20198
**High Alcohol Intake**				
Men	-0.7 (-1.8,0.1)	24077	0.0 (-0.9,0.8)	22609
Women	0.3 (-0.6,1.4)	22004	0.4 (-0.5,1.5)	20198
**Hypertension**				
Men	-1.6 (-3.3,-0.8)	25710	-0.5 (-1.3,0.1)	22609
Women	-2.1 (-3.6,-1.3)	24094	-0.6 (-1.4,0.1)	20198
**Diabetes**				
Men	-4.9 (-10.0,-3.1)	25696	-2.8 (-5.4,-1.5)	22609
Women	-6.1 (-10.6,-4.0)	24087	-2.4 (-5.1,-0.8)	20198
**Physical Inactivity**				
Men	-5.1 (-9.5,-3.3)	24366	-3.5 (-6.2,-2.3)	22609
Women	-5.2 (-8.9,-3.6)	22635	-4.3 (-7.7,-2.9)	20198

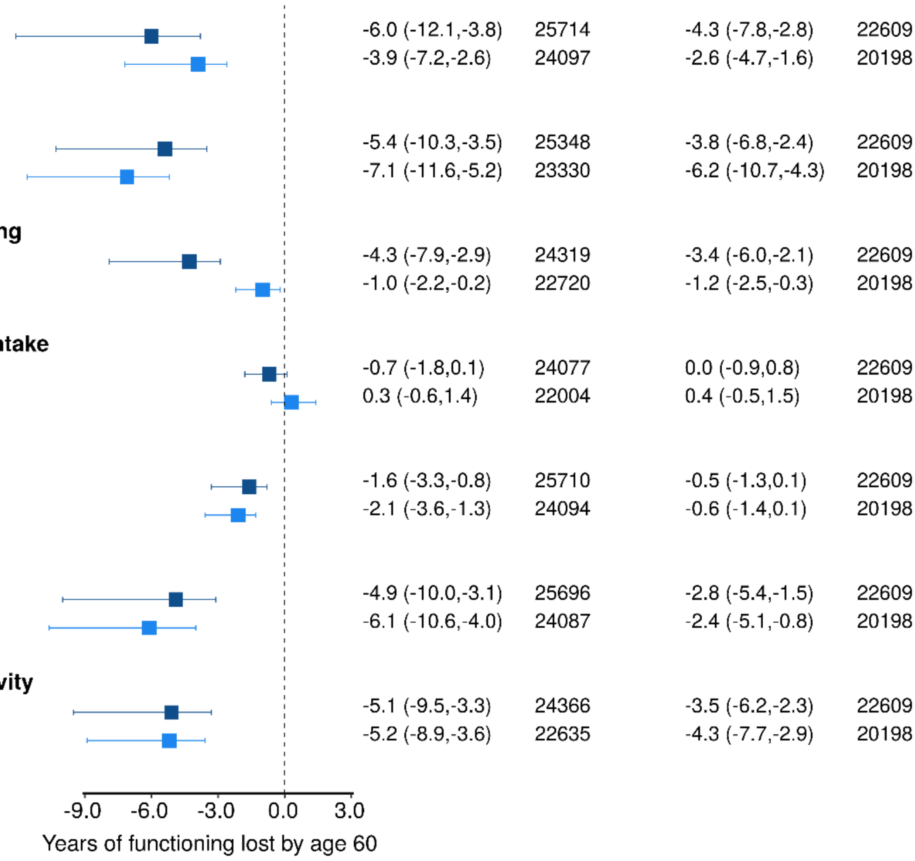

Figure S8. Years of functioning lost by 60 years of age due to suboptimal risk factors including participants walking with a mobility aid

The minimally adjusted models were only adjusted for age, age^2, height, year of birth and walked distances; in the mutually adjusted models, SES and the non-communicable diseases risk factors are mutually adjusted.

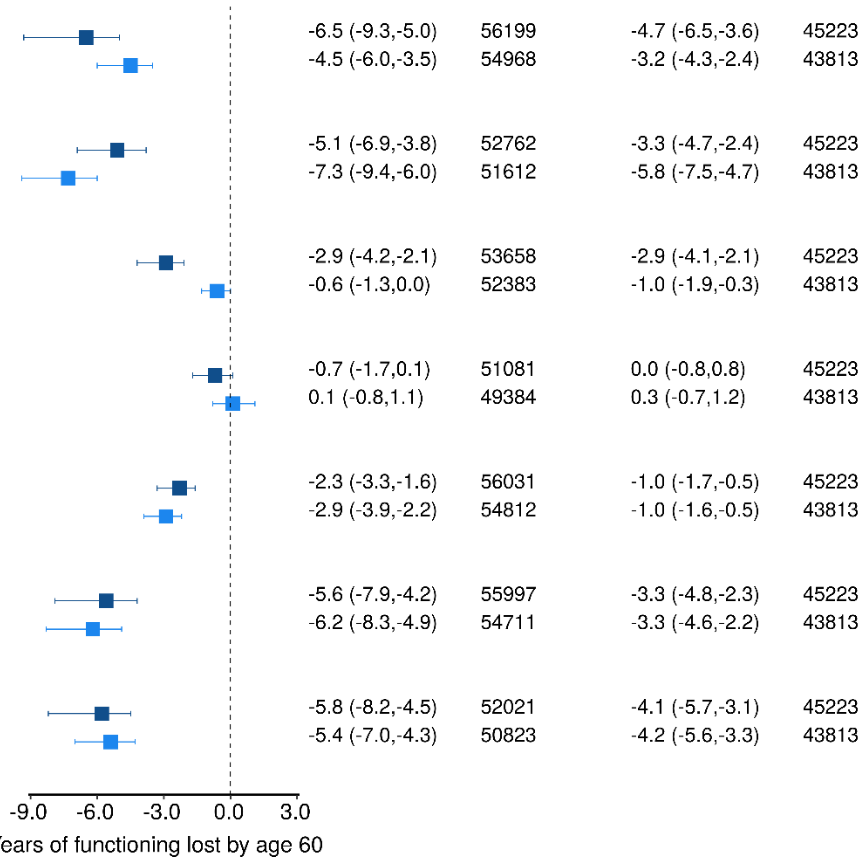

Risk factor	Minimally adjusted YFL (95% CI)	Minimally adjusted N	Mutually adjusted YFL (95% CI)	Mutually adjusted N
**Low SES**				
Men	-6.5 (-9.3,-5.0)	56199	-4.7 (-6.5,-3.6)	45223
Women	-4.5 (-6.0,-3.5)	54968	-3.2 (-4.3,-2.4)	43813
**Obesity**				
Men	-5.1 (-6.9,-3.8)	52762	-3.3 (-4.7,-2.4)	45223
Women	-7.3 (-9.4,-6.0)	51612	-5.8 (-7.5,-4.7)	43813
**Current Smoking**				
Men	-2.9 (-4.2,-2.1)	53658	-2.9 (-4.1,-2.1)	45223
Women	-0.6 (-1.3,0.0)	52383	-1.0 (-1.9,-0.3)	43813
**High Alcohol Intake**				
Men	-0.7 (-1.7,0.1)	51081	0.0 (-0.8,0.8)	45223
Women	0.1 (-0.8,1.1)	49384	0.3 (-0.7,1.2)	43813
**Hypertension**				
Men	-2.3 (-3.3,-1.6)	56031	-1.0 (-1.7,-0.5)	45223
Women	-2.9 (-3.9,-2.2)	54812	-1.0 (-1.6,-0.5)	43813
**Diabetes**				
Men	-5.6 (-7.9,-4.2)	55997	-3.3 (-4.8,-2.3)	45223
Women	-6.2 (-8.3,-4.9)	54711	-3.3 (-4.6,-2.2)	43813
**Physical Inactivity**				
Men	-5.8 (-8.2,-4.5)	52021	-4.1 (-5.7,-3.1)	45223
Women	-5.4 (-7.0,-4.3)	50823	-4.2 (-5.6,-3.3)	43813

Appendix 4. Years of functioning lost by 60 years of age in single studies

Study-specific YFL estimates were obtained from minimally-adjusted models and meta-analysed using the Hartung-Knapp random-effects method. Study-specific YFLs are represented with a gray square, which size is proportional to the weight estimated by the random-effects method (rightmost column). To assess heterogeneity between studies, we computed I^2 and τ^2 statistics; I^2 assesses heterogeneity attributable to variation in the true association and τ^2 measures the inter-cohort variance. To account for τ^2 in the uncertainty around the pooled estimates, we further calculated 95% prediction intervals for YFL. Pooled YFL is represented with a grey diamond and the 95% prediction interval with a black bar.

Low SES − men

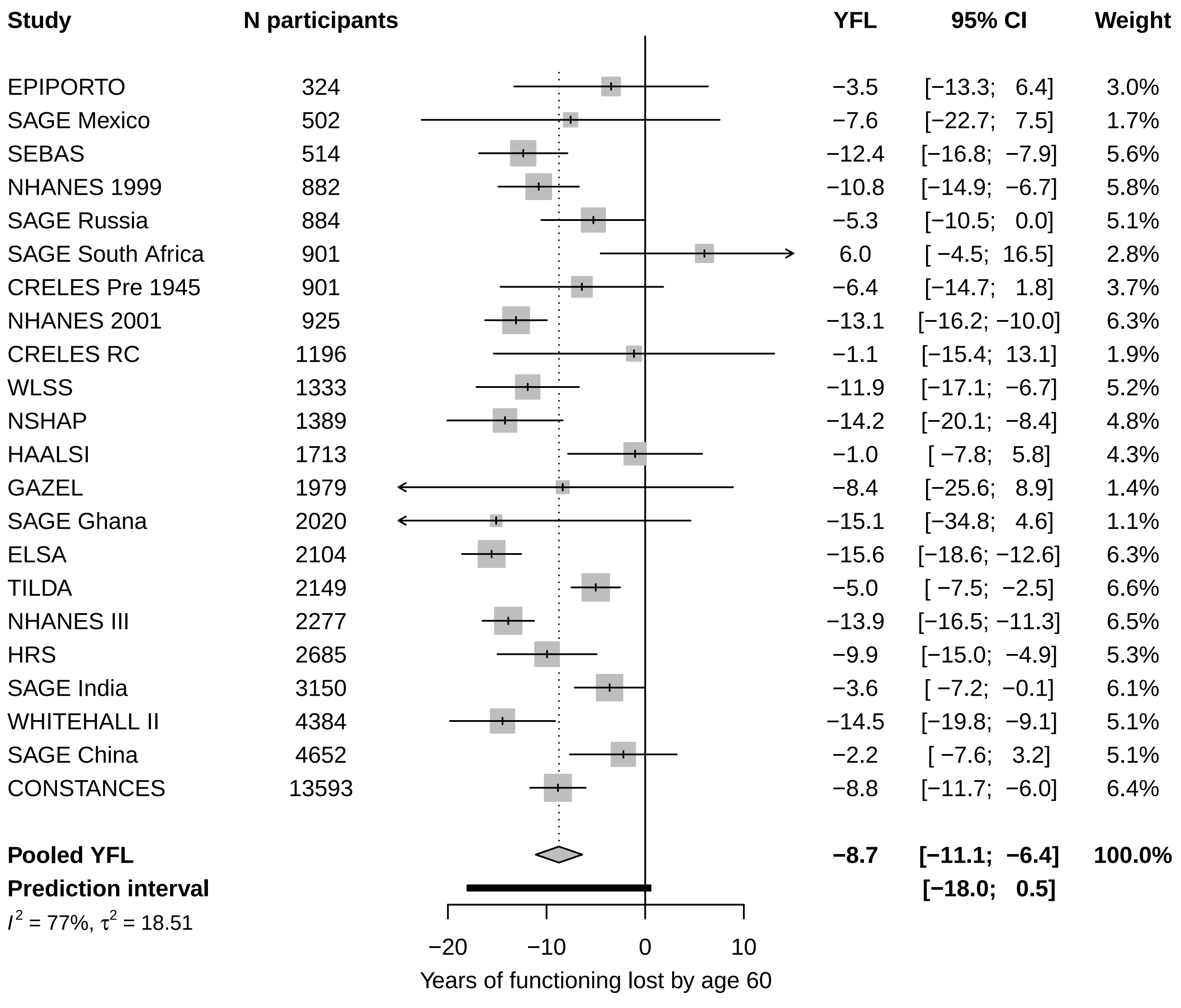
Study N participants YFL 95% CI Weight
EPIPORTO 324 −3.5 [−13.3; 6.4] 3.0%
SAGE Mexico 502 −7.6 [−22.7; 7.5] 1.7%
SEBAS 514 −12.4 [−16.8; −7.9] 5.6%
NHANES 1999 882 −10.8 [−14.9; −6.7] 5.8%
SAGE Russia 884 −5.3 [−10.5; 0.0] 5.1%
SAGE South Africa 901 6.0 [−4.5; 16.5] 2.8%
CRELES Pre 1945 901 −6.4 [−14.7; 1.8] 3.7%
NHANES 2001 925 −13.1 [−16.2; −10.0] 6.3%
CRELES RC 1196 −1.1 [−15.4; 13.1] 1.9%
WLSS 1333 −11.9 [−17.1; −6.7] 5.2%
NSHAP 1389 −14.2 [−20.1; −8.4] 4.8%
HAALSI 1713 −1.0 [−7.8; 5.8] 4.3%
GAZEL 1979 −8.4 [−25.6; 8.9] 1.4%
SAGE Ghana 2020 −15.1 [−34.8; 4.6] 1.1%
ELSA 2104 −15.6 [−18.6; −12.6] 6.3%
TILDA 2149 −5.0 [−7.5; −2.5] 6.6%
NHANES III 2277 −13.9 [−16.5; −11.3] 6.5%
HRS 2685 −9.9 [−15.0; −4.9] 5.3%
SAGE India 3150 −3.6 [−7.2; −0.1] 6.1%
WHITEHALL II 4384 −14.5 [−19.8; −9.1] 5.1%
SAGE China 4652 −2.2 [−7.6; 3.2] 5.1%
CONSTANCES 13593 −8.8 [−11.7; −6.0] 6.4%
Pooled YFL −8.7 [−11.1; −6.4] 100.0%
Prediction interval [−18.0; 0.5]
I^2 = 77%, τ^2 = 18.51
−20 −10 0 10
Years of functioning lost by age 60

Low SES − women

Study	N participants	YFL	95% CI	Weight
SEBAS	331	−4.5	[−10.0; 1.1]	4.7%
SAGE Mexico	370	−8.9	[−13.6; −4.2]	5.1%
EPIPORTO	478	−0.9	[−5.2; 3.3]	5.3%
GAZEL	603	−12.5	[−34.2; 9.3]	0.9%
CRELES Pre 1945	702	−6.8	[−12.1; −1.5]	4.8%
NHANES 1999	801	−10.4	[−15.2; −5.7]	5.0%
NHANES 2001	871	−13.0	[−17.2; −8.8]	5.3%
SAGE South Africa	1159	−14.9	[−34.5; 4.6]	1.1%
WLSS	1465	−6.8	[−16.2; 2.6]	3.1%
SAGE Russia	1570	−6.3	[−11.3; −1.3]	4.9%
NSHAP	1626	−14.6	[−18.3; −10.8]	5.5%
SAGE India	1661	−4.9	[−18.4; 8.7]	2.0%
CRELES RC	1766	−0.6	[−3.4; 2.3]	5.8%
SAGE Ghana	1804	−7.1	[−17.1; 2.9]	2.9%
WHITEHALL II	1809	−9.1	[−12.2; −6.1]	5.8%
HAALSI	1997	5.6	[0.6; 10.6]	4.9%
NHANES III	2184	−16.6	[−20.0; −13.2]	5.6%
ELSA	2545	−9.1	[−12.9; −5.4]	5.5%
TILDA	2638	−6.8	[−9.1; −4.4]	6.0%
HRS	3110	−7.5	[−12.6; −2.5]	4.9%
SAGE China	4786	−4.8	[−8.1; −1.5]	5.7%
CONSTANCES	14819	−7.6	[−11.7; −3.4]	5.3%
**Pooled YFL**		**−7.3**	**[−9.6; −5.0]**	**100.0%**
**Prediction interval**			**[−17.2; 2.6]**	

I^2 = 82%, τ^2 = 21.19

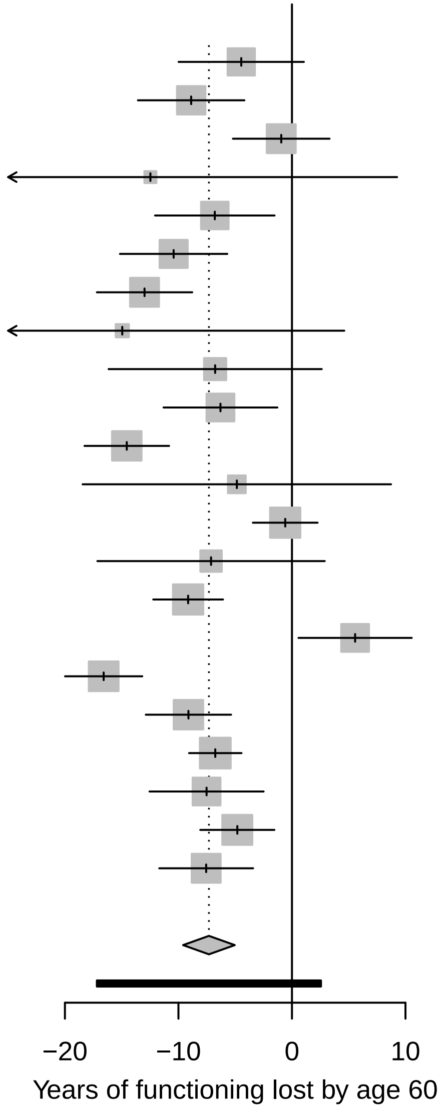

Obesity − men

Study	N participants	YFL	95% CI	Weight
EPIPORTO	323	−11.2	[−18.4; −3.9]	4.1%
SAGE Mexico	497	1.2	[−12.2; 14.5]	1.3%
SEBAS	503	−6.8	[−20.4; 6.8]	1.2%
CRELES Pre 1945	830	2.0	[−23.4; 27.4]	0.4%
SAGE South Africa	856	−5.5	[−13.8; 2.8]	3.1%
SAGE Russia	862	−3.7	[−13.2; 5.9]	2.4%
NHANES 1999	875	−3.0	[−10.9; 4.8]	3.5%
NHANES 2001	909	−6.3	[−12.1; −0.5]	6.2%
WLSS	1093	−11.4	[−17.5; −5.4]	5.7%
CRELES RC	1176	1.5	[−15.8; 18.8]	0.8%
NSHAP	1360	−5.2	[−9.7; −0.7]	9.6%
HAALSI	1564	−3.1	[−9.9; 3.7]	4.6%
SAGE Ghana	1724	−10.3	[−22.4; 1.8]	1.5%
GAZEL	1945	−20.3	[−36.1; −4.4]	0.9%
SAGE India	2026	−7.0	[−14.7; 0.7]	3.6%
ELSA	2069	−9.8	[−13.5; −6.1]	13.2%
TILDA	2140	−9.6	[−12.8; −6.3]	15.9%
NHANES III	2229	−5.9	[−15.5; 3.7]	2.4%
HRS	2446	−1.1	[−11.7; 9.5]	2.0%
WHITEHALL II	4358	−9.5	[−13.4; −5.6]	12.2%
SAGE China	4427	2.7	[−8.1; 13.5]	1.9%
CONSTANCES	13344	−10.3	[−18.1; −2.6]	3.6%
**Pooled YFL**		**−7.6**	**[−9.2; −6.0]**	**100.0%**
**Prediction interval**			**[−10.2; −5.0]**	

$I^2 = 8\%$, $\tau^2 = 0.9455$

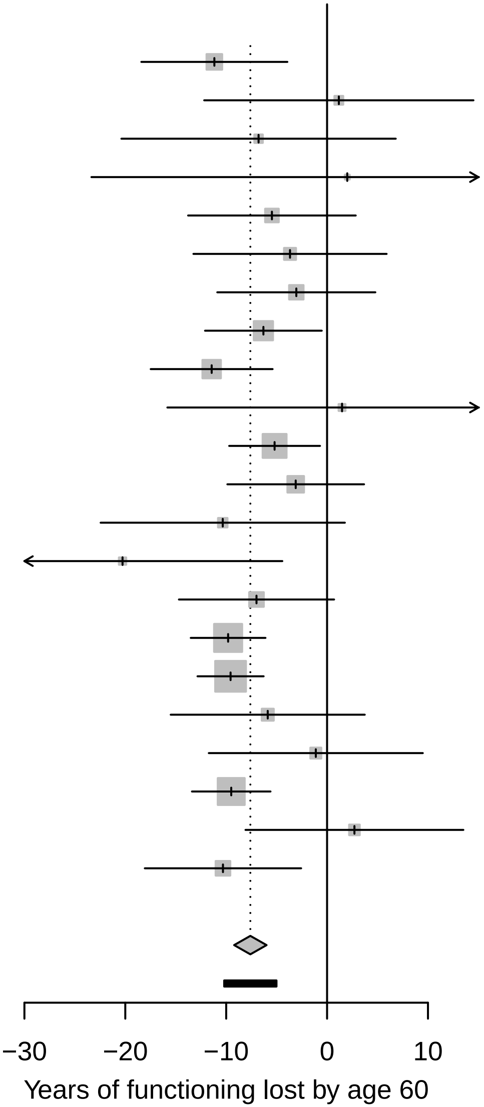

Obesity − women

Study	N participants	YFL	95% CI	Weight
SEBAS	323	5.1	[−4.0; 14.1]	3.1%
SAGE Mexico	361	4.9	[−9.5; 19.2]	1.5%
EPIPORTO	476	−12.1	[−20.3; −3.9]	3.5%
GAZEL	584	−13.7	[−25.7; −1.7]	2.1%
CRELES Pre 1945	658	−1.7	[−17.1; 13.6]	1.4%
NHANES 1999	790	−14.4	[−17.9; −10.9]	6.9%
NHANES 2001	844	−13.8	[−17.7; −9.9]	6.5%
SAGE India	1044	−4.9	[−14.9; 5.1]	2.7%
SAGE South Africa	1117	−37.2	[−59.0; −15.4]	0.7%
WLSS	1205	−12.2	[−15.3; −9.1]	7.2%
SAGE Russia	1528	−6.5	[−11.7; −1.4]	5.5%
SAGE Ghana	1537	−7.7	[−15.1; −0.3]	3.9%
NSHAP	1582	−10.9	[−15.0; −6.9]	6.4%
CRELES RC	1738	−4.5	[−15.3; 6.4]	2.4%
WHITEHALL II	1776	−12.4	[−20.1; −4.7]	3.8%
HAALSI	1944	−5.9	[−12.2; 0.5]	4.6%
NHANES III	2130	−16.1	[−18.4; −13.8]	7.9%
ELSA	2486	−11.0	[−13.6; −8.4]	7.6%
TILDA	2610	−11.3	[−13.7; −8.9]	7.8%
HRS	2797	−8.9	[−12.9; −4.8]	6.4%
SAGE China	4558	−7.0	[−10.1; −3.8]	7.2%
CONSTANCES	14223	−26.8	[−48.0; −5.6]	0.8%
**Pooled YFL**		**−10.2**	**[−12.6; −7.7]**	**100.0%**
**Prediction interval**			**[−17.6; −2.7]**	

$I^2 = 69\%$, $\tau^2 = 11.45$

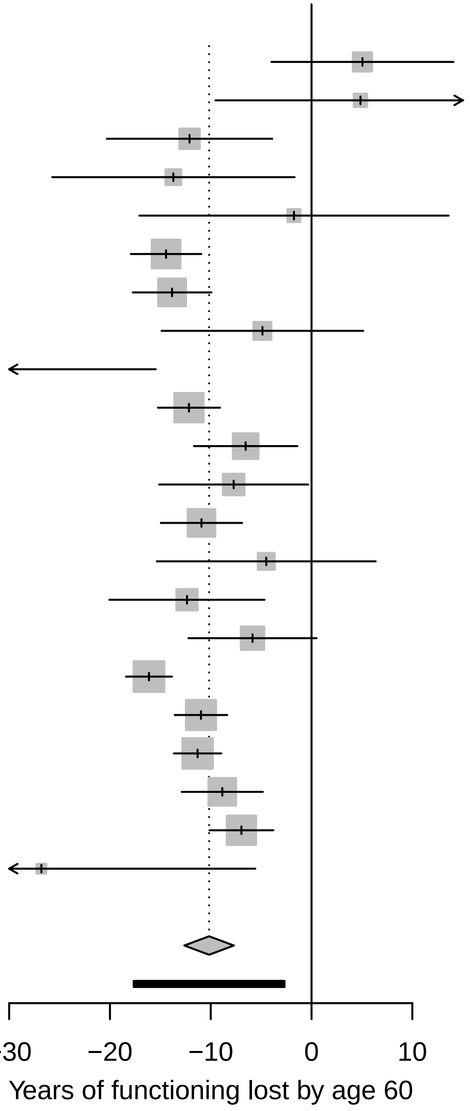

Current smoking − men

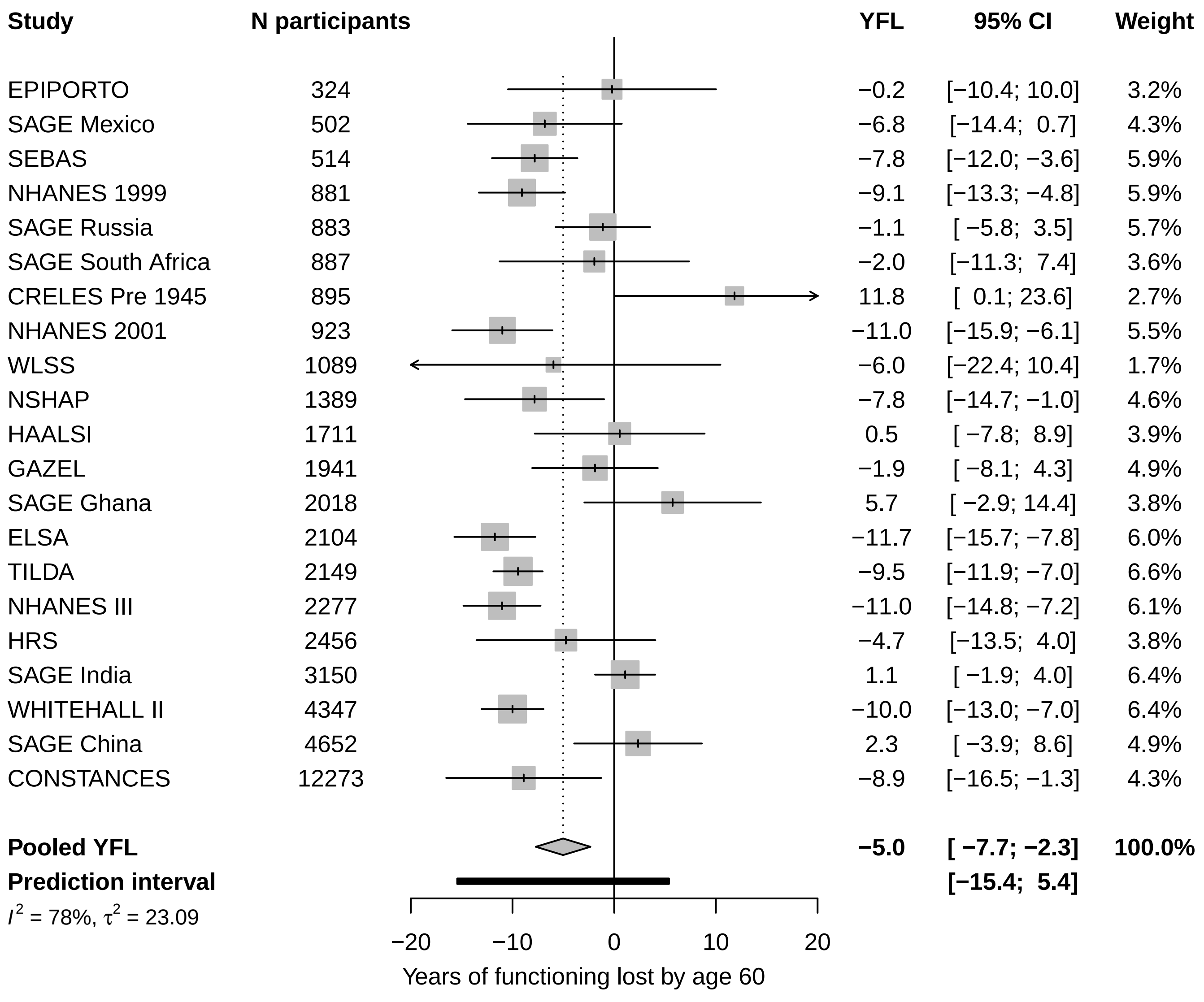

Current smoking − women

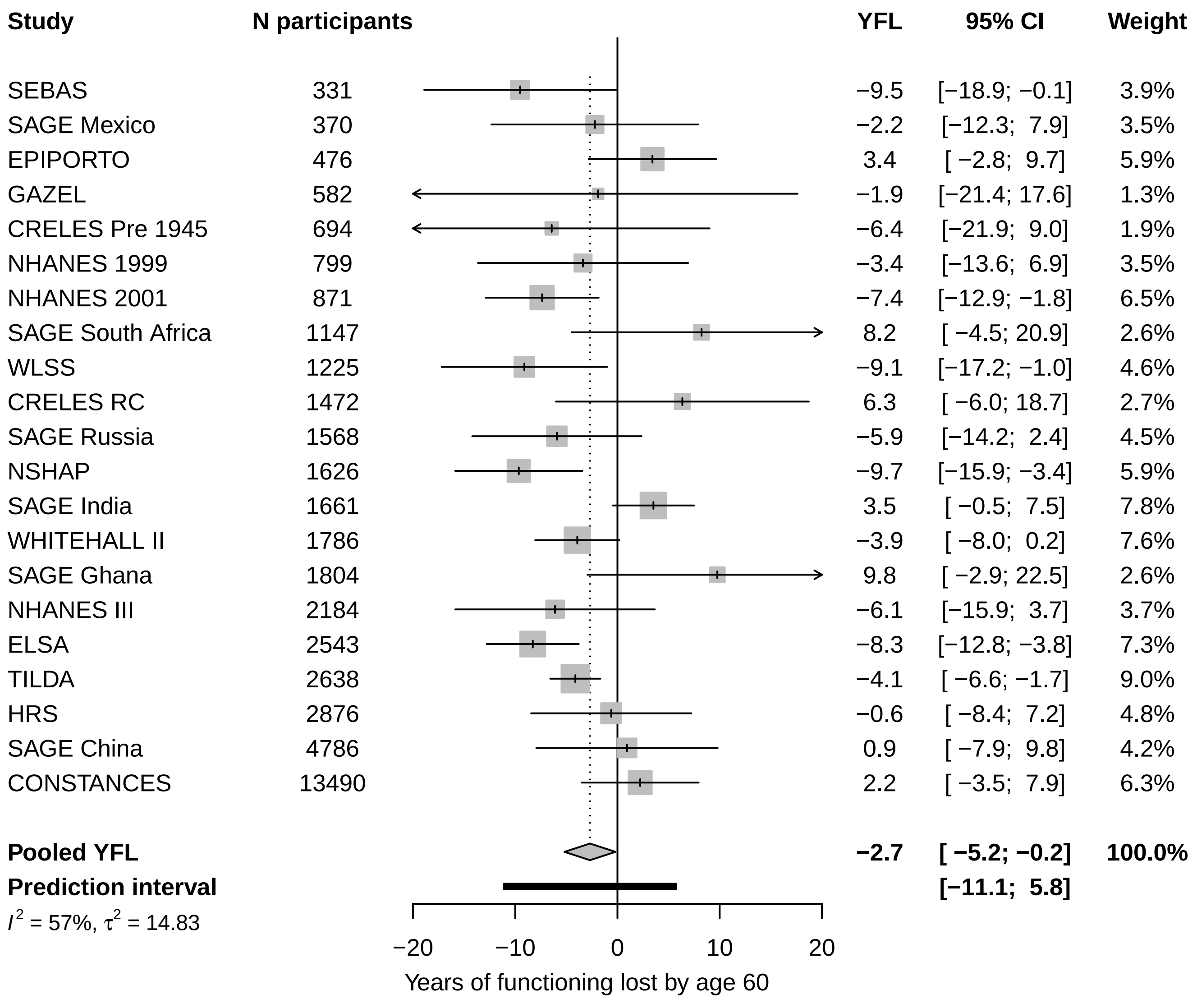

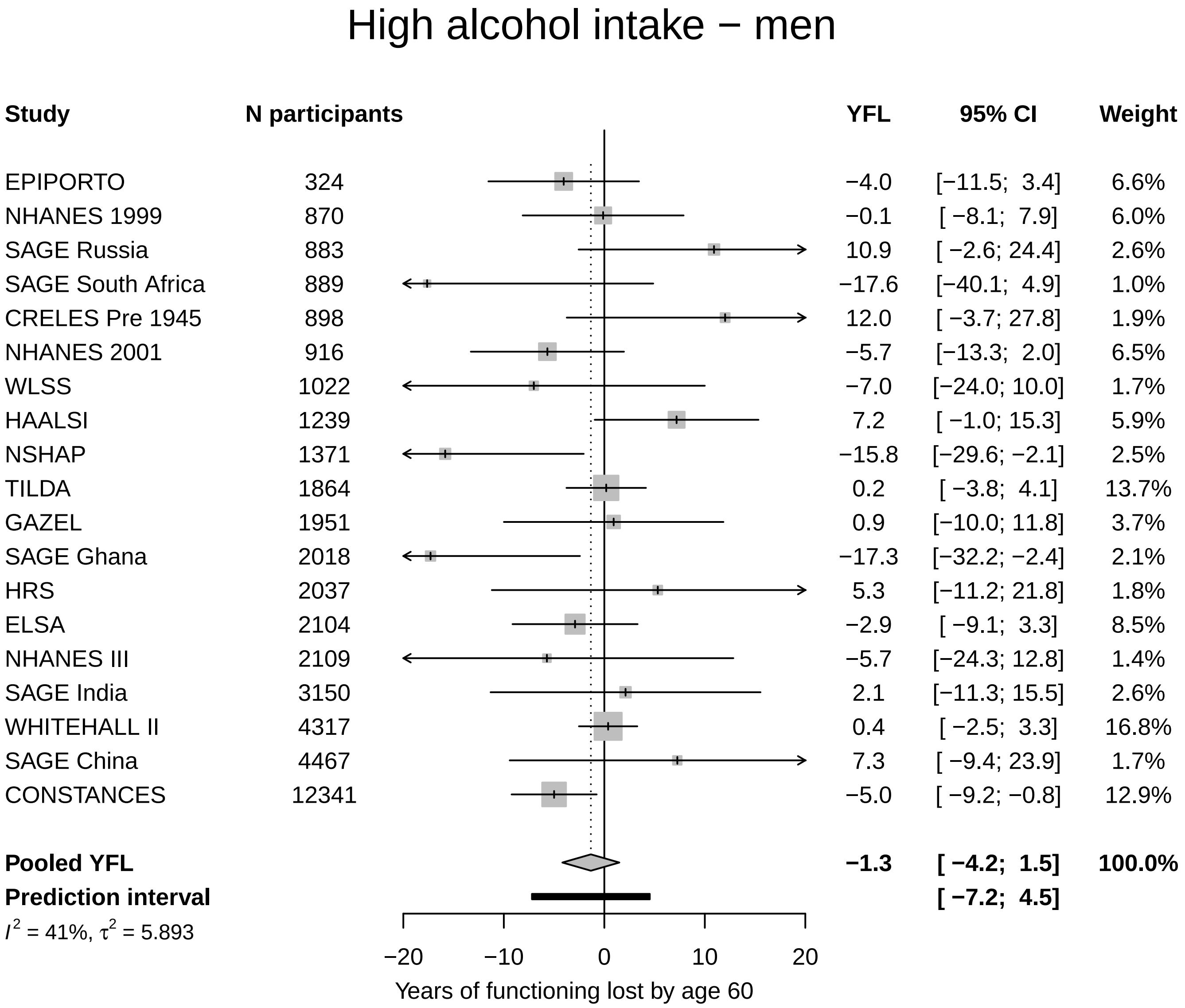
High alcohol intake − men
Study
N participants
YFL
95% CI
Weight
EPIPORTO 324 −4.0 [−11.5; 3.4] 6.6%
NHANES 1999 870 −0.1 [−8.1; 7.9] 6.0%
SAGE Russia 883 10.9 [−2.6; 24.4] 2.6%
SAGE South Africa 889 −17.6 [−40.1; 4.9] 1.0%
CRELES Pre 1945 898 12.0 [−3.7; 27.8] 1.9%
NHANES 2001 916 −5.7 [−13.3; 2.0] 6.5%
WLSS 1022 −7.0 [−24.0; 10.0] 1.7%
HAALSI 1239 7.2 [−1.0; 15.3] 5.9%
NSHAP 1371 −15.8 [−29.6; −2.1] 2.5%
TILDA 1864 0.2 [−3.8; 4.1] 13.7%
GAZEL 1951 0.9 [−10.0; 11.8] 3.7%
SAGE Ghana 2018 −17.3 [−32.2; −2.4] 2.1%
HRS 2037 5.3 [−11.2; 21.8] 1.8%
ELSA 2104 −2.9 [−9.1; 3.3] 8.5%
NHANES III 2109 −5.7 [−24.3; 12.8] 1.4%
SAGE India 3150 2.1 [−11.3; 15.5] 2.6%
WHITEHALL II 4317 0.4 [−2.5; 3.3] 16.8%
SAGE China 4467 7.3 [−9.4; 23.9] 1.7%
CONSTANCES 12341 −5.0 [−9.2; −0.8] 12.9%
Pooled YFL −1.3 [−4.2; 1.5] 100.0%
Prediction interval [−7.2; 4.5]
I² = 41%, τ² = 5.893
−20
−10
0
10
20
Years of functioning lost by age 60

High alcohol intake − women

Study	N participants	YFL	95% CI	Weight
EPIPORTO	478	−1.0	[−7.4; 5.5]	11.3%
GAZEL	595	−6.4	[−28.4; 15.6]	1.7%
NHANES 1999	791	−3.3	[−16.8; 10.1]	4.0%
NHANES 2001	857	3.5	[−8.0; 15.0]	5.2%
WLSS	993	1.0	[−23.4; 25.5]	1.4%
NSHAP	1608	−4.0	[−19.9; 11.9]	3.0%
HAALSI	1704	11.4	[0.8; 22.1]	5.8%
WHITEHALL II	1759	5.4	[0.8; 9.9]	15.5%
SAGE Ghana	1804	−12.6	[−30.7; 5.5]	2.4%
NHANES III	2025	−18.4	[−33.7; −3.1]	3.2%
TILDA	2318	−2.6	[−6.5; 1.3]	17.1%
HRS	2339	−0.9	[−16.3; 14.5]	3.2%
ELSA	2545	−0.5	[−8.3; 7.4]	9.0%
SAGE China	4748	−5.1	[−26.3; 16.2]	1.8%
CONSTANCES	13104	−2.1	[−6.6; 2.5]	15.5%
**Pooled YFL**		**−0.6**	**[−3.8; 2.6]**	**100.0%**
**Prediction interval**			**[−7.9; 6.7]**	

I^2 = 34%, τ^2 = 9.221

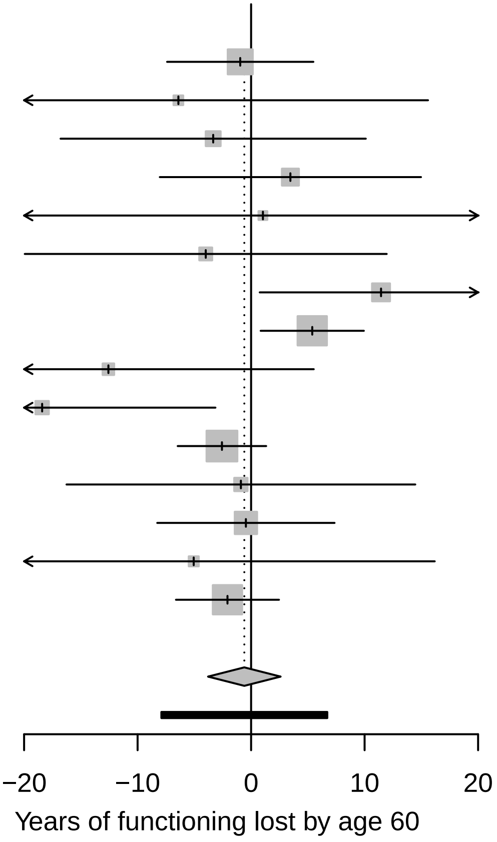

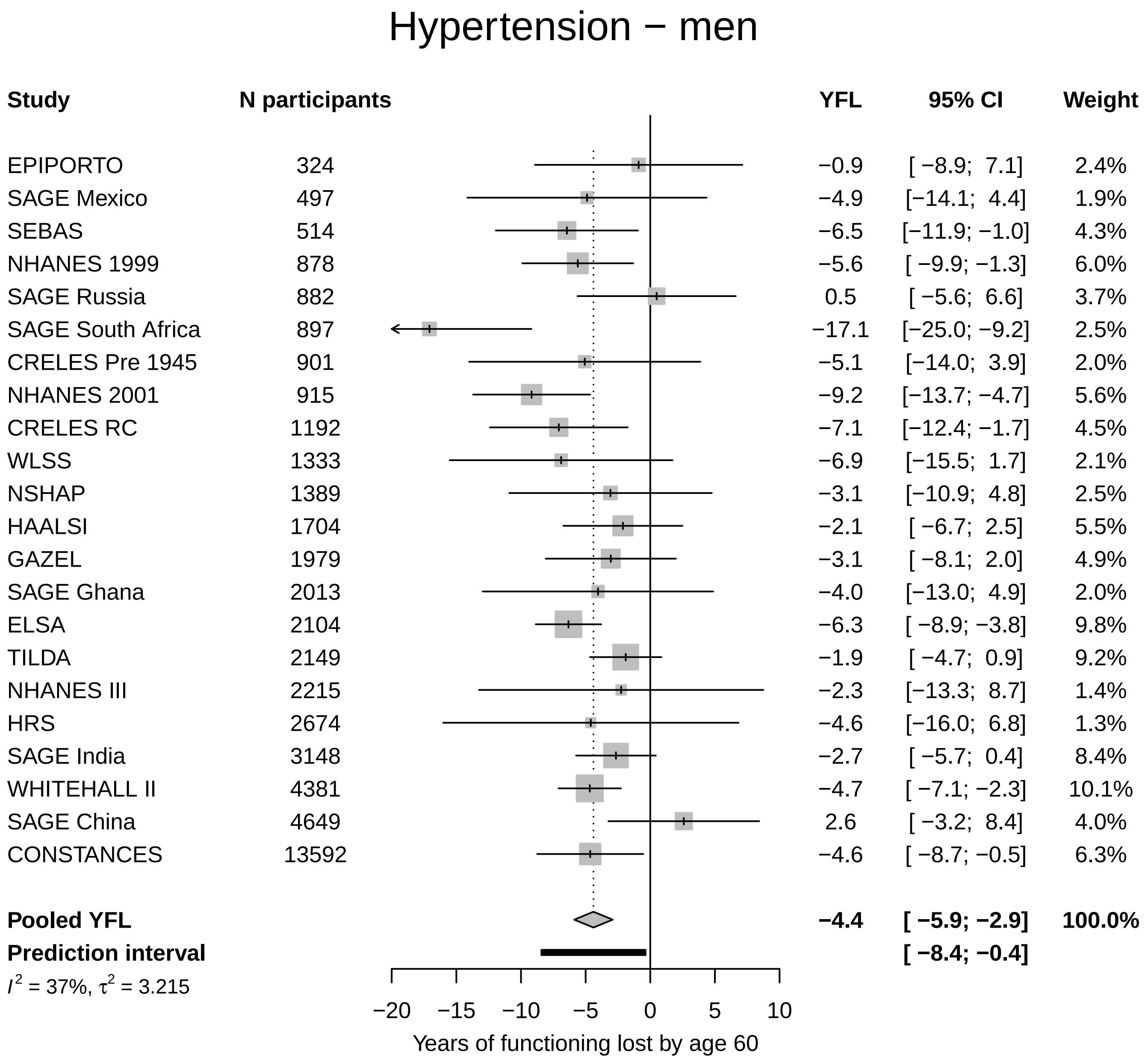
Hypertension − men
Study
N participants
YFL
95% CI
Weight
EPIPORTO 324 −0.9 [−8.9; 7.1] 2.4%
SAGE Mexico 497 −4.9 [−14.1; 4.4] 1.9%
SEBAS 514 −6.5 [−11.9; −1.0] 4.3%
NHANES 1999 878 −5.6 [−9.9; −1.3] 6.0%
SAGE Russia 882 0.5 [−5.6; 6.6] 3.7%
SAGE South Africa 897 −17.1 [−25.0; −9.2] 2.5%
CRELES Pre 1945 901 −5.1 [−14.0; 3.9] 2.0%
NHANES 2001 915 −9.2 [−13.7; −4.7] 5.6%
CRELES RC 1192 −7.1 [−12.4; −1.7] 4.5%
WLSS 1333 −6.9 [−15.5; 1.7] 2.1%
NSHAP 1389 −3.1 [−10.9; 4.8] 2.5%
HAALSI 1704 −2.1 [−6.7; 2.5] 5.5%
GAZEL 1979 −3.1 [−8.1; 2.0] 4.9%
SAGE Ghana 2013 −4.0 [−13.0; 4.9] 2.0%
ELSA 2104 −6.3 [−8.9; −3.8] 9.8%
TILDA 2149 −1.9 [−4.7; 0.9] 9.2%
NHANES III 2215 −2.3 [−13.3; 8.7] 1.4%
HRS 2674 −4.6 [−16.0; 6.8] 1.3%
SAGE India 3148 −2.7 [−5.7; 0.4] 8.4%
WHITEHALL II 4381 −4.7 [−7.1; −2.3] 10.1%
SAGE China 4649 2.6 [−3.2; 8.4] 4.0%
CONSTANCES 13592 −4.6 [−8.7; −0.5] 6.3%
Pooled YFL −4.4 [−5.9; −2.9] 100.0%
Prediction interval [−8.4; −0.4]
I^2 = 37%, τ^2 = 3.215
−20 −15 −10 −5 0 5 10
Years of functioning lost by age 60

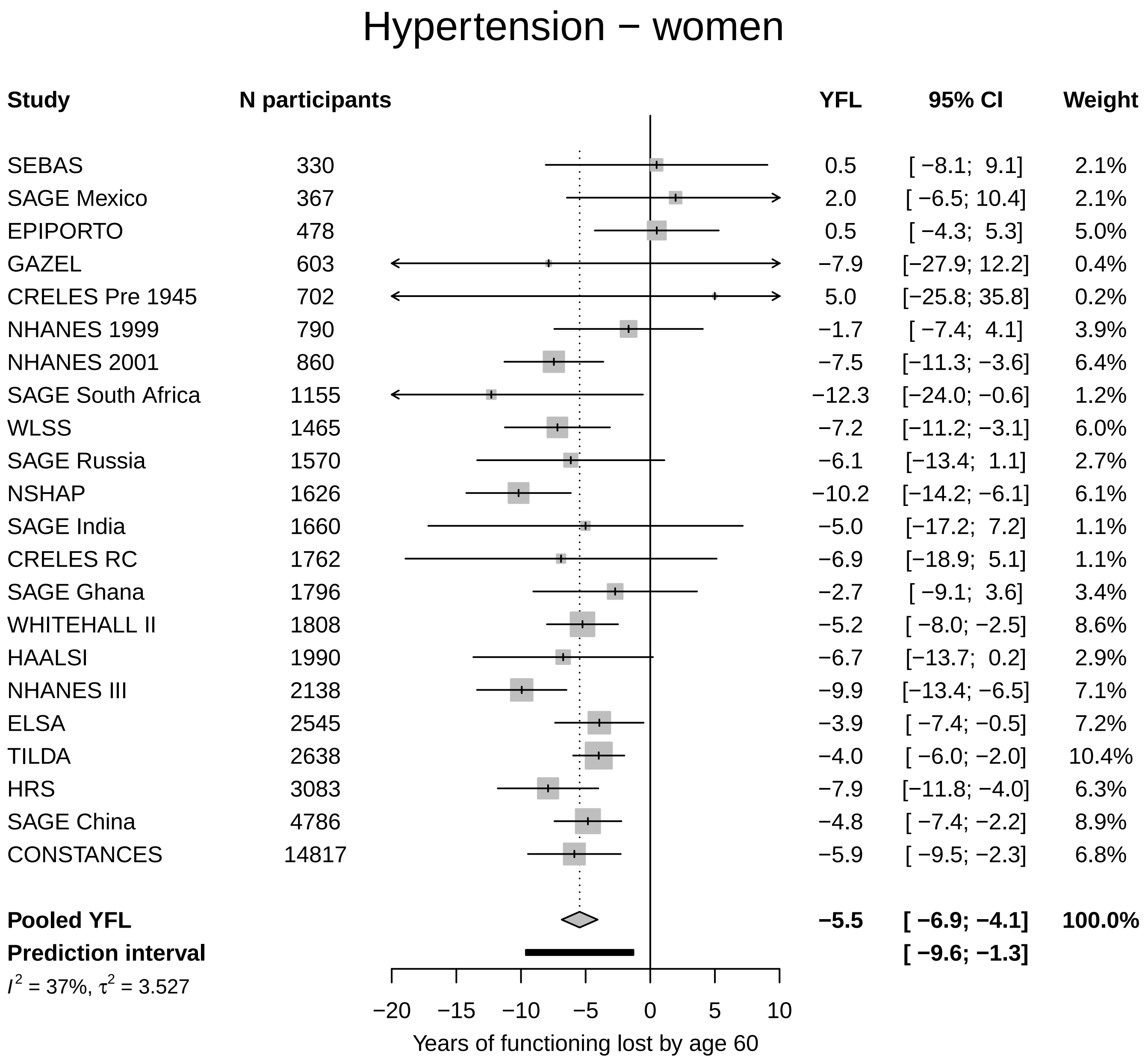
Hypertension − women
Study
N participants
YFL
95% CI
Weight
SEBAS 330 0.5 [−8.1; 9.1] 2.1%
SAGE Mexico 367 2.0 [−6.5; 10.4] 2.1%
EPIPORTO 478 0.5 [−4.3; 5.3] 5.0%
GAZEL 603 −7.9 [−27.9; 12.2] 0.4%
CRELES Pre 1945 702 5.0 [−25.8; 35.8] 0.2%
NHANES 1999 790 −1.7 [−7.4; 4.1] 3.9%
NHANES 2001 860 −7.5 [−11.3; −3.6] 6.4%
SAGE South Africa 1155 −12.3 [−24.0; −0.6] 1.2%
WLSS 1465 −7.2 [−11.2; −3.1] 6.0%
SAGE Russia 1570 −6.1 [−13.4; 1.1] 2.7%
NSHAP 1626 −10.2 [−14.2; −6.1] 6.1%
SAGE India 1660 −5.0 [−17.2; 7.2] 1.1%
CRELES RC 1762 −6.9 [−18.9; 5.1] 1.1%
SAGE Ghana 1796 −2.7 [−9.1; 3.6] 3.4%
WHITEHALL II 1808 −5.2 [−8.0; −2.5] 8.6%
HAALSI 1990 −6.7 [−13.7; 0.2] 2.9%
NHANES III 2138 −9.9 [−13.4; −6.5] 7.1%
ELSA 2545 −3.9 [−7.4; −0.5] 7.2%
TILDA 2638 −4.0 [−6.0; −2.0] 10.4%
HRS 3083 −7.9 [−11.8; −4.0] 6.3%
SAGE China 4786 −4.8 [−7.4; −2.2] 8.9%
CONSTANCES 14817 −5.9 [−9.5; −2.3] 6.8%
Pooled YFL −5.5 [−6.9; −4.1] 100.0%
Prediction interval [−9.6; −1.3]
I² = 37%, τ² = 3.527
−20 −15 −10 −5 0 5 10
Years of functioning lost by age 60

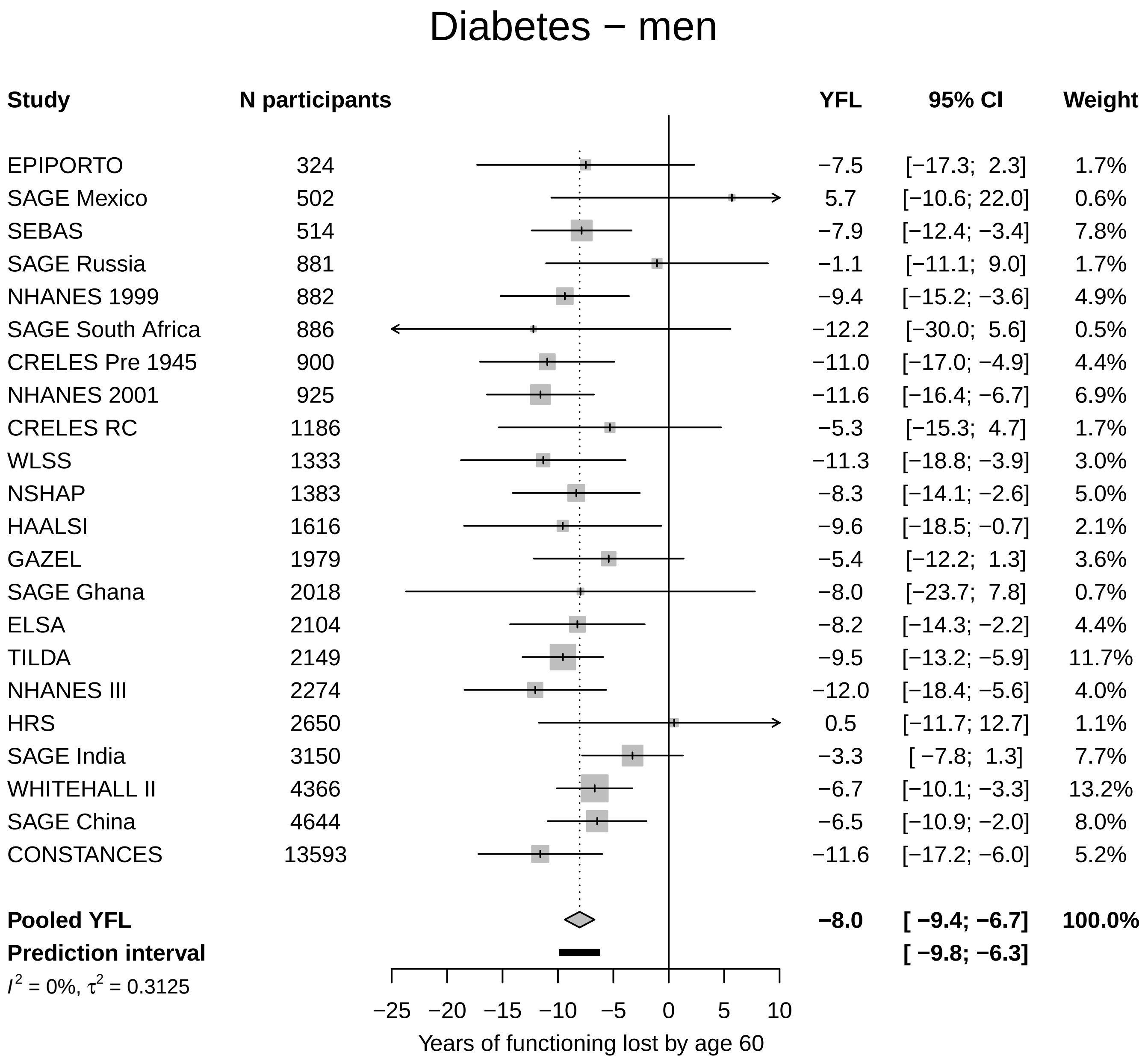
Diabetes − men
Study
N participants
YFL
95% CI
Weight
EPIPORTO 324 −7.5 [−17.3; 2.3] 1.7%
SAGE Mexico 502 5.7 [−10.6; 22.0] 0.6%
SEBAS 514 −7.9 [−12.4; −3.4] 7.8%
SAGE Russia 881 −1.1 [−11.1; 9.0] 1.7%
NHANES 1999 882 −9.4 [−15.2; −3.6] 4.9%
SAGE South Africa 886 −12.2 [−30.0; 5.6] 0.5%
CRELES Pre 1945 900 −11.0 [−17.0; −4.9] 4.4%
NHANES 2001 925 −11.6 [−16.4; −6.7] 6.9%
CRELES RC 1186 −5.3 [−15.3; 4.7] 1.7%
WLSS 1333 −11.3 [−18.8; −3.9] 3.0%
NSHAP 1383 −8.3 [−14.1; −2.6] 5.0%
HAALSI 1616 −9.6 [−18.5; −0.7] 2.1%
GAZEL 1979 −5.4 [−12.2; 1.3] 3.6%
SAGE Ghana 2018 −8.0 [−23.7; 7.8] 0.7%
ELSA 2104 −8.2 [−14.3; −2.2] 4.4%
TILDA 2149 −9.5 [−13.2; −5.9] 11.7%
NHANES III 2274 −12.0 [−18.4; −5.6] 4.0%
HRS 2650 0.5 [−11.7; 12.7] 1.1%
SAGE India 3150 −3.3 [−7.8; 1.3] 7.7%
WHITEHALL II 4366 −6.7 [−10.1; −3.3] 13.2%
SAGE China 4644 −6.5 [−10.9; −2.0] 8.0%
CONSTANCES 13593 −11.6 [−17.2; −6.0] 5.2%
Pooled YFL −8.0 [−9.4; −6.7] 100.0%
Prediction interval [−9.8; −6.3]
I^2 = 0%, τ^2 = 0.3125
−25 −20 −15 −10 −5 0 5 10
Years of functioning lost by age 60

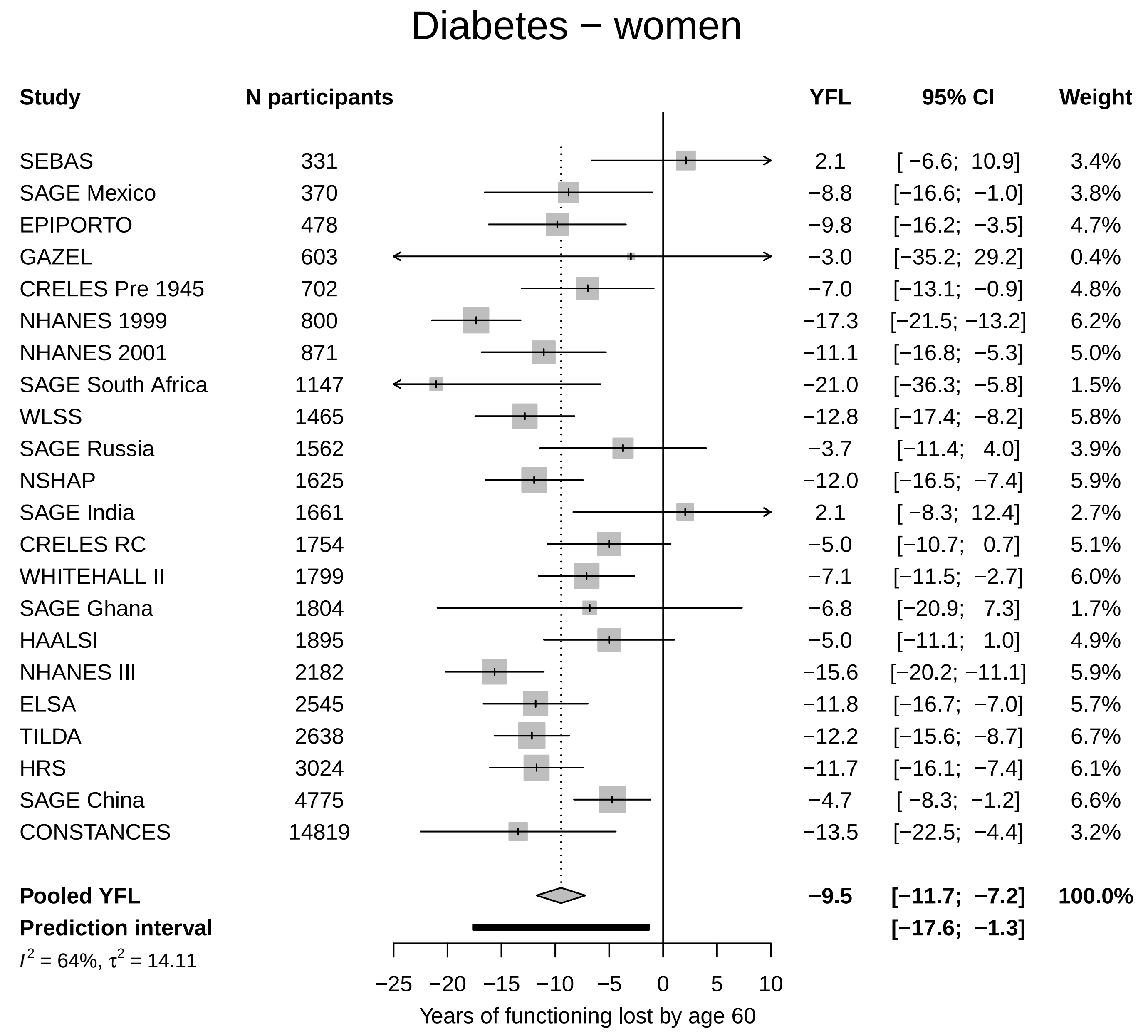
Diabetes − women
Study
N participants
YFL
95% CI
Weight
SEBAS 331 2.1 [−6.6; 10.9] 3.4%
SAGE Mexico 370 −8.8 [−16.6; −1.0] 3.8%
EPIPORTO 478 −9.8 [−16.2; −3.5] 4.7%
GAZEL 603 −3.0 [−35.2; 29.2] 0.4%
CRELES Pre 1945 702 −7.0 [−13.1; −0.9] 4.8%
NHANES 1999 800 −17.3 [−21.5; −13.2] 6.2%
NHANES 2001 871 −11.1 [−16.8; −5.3] 5.0%
SAGE South Africa 1147 −21.0 [−36.3; −5.8] 1.5%
WLSS 1465 −12.8 [−17.4; −8.2] 5.8%
SAGE Russia 1562 −3.7 [−11.4; 4.0] 3.9%
NSHAP 1625 −12.0 [−16.5; −7.4] 5.9%
SAGE India 1661 2.1 [−8.3; 12.4] 2.7%
CRELES RC 1754 −5.0 [−10.7; 0.7] 5.1%
WHITEHALL II 1799 −7.1 [−11.5; −2.7] 6.0%
SAGE Ghana 1804 −6.8 [−20.9; 7.3] 1.7%
HAALSI 1895 −5.0 [−11.1; 1.0] 4.9%
NHANES III 2182 −15.6 [−20.2; −11.1] 5.9%
ELSA 2545 −11.8 [−16.7; −7.0] 5.7%
TILDA 2638 −12.2 [−15.6; −8.7] 6.7%
HRS 3024 −11.7 [−16.1; −7.4] 6.1%
SAGE China 4775 −4.7 [−8.3; −1.2] 6.6%
CONSTANCES 14819 −13.5 [−22.5; −4.4] 3.2%
Pooled YFL −9.5 [−11.7; −7.2] 100.0%
Prediction interval [−17.6; −1.3]
I^2 = 64%, τ^2 = 14.11
−25 −20 −15 −10 −5 0 5 10
Years of functioning lost by age 60

Physical Inactivity − men

Study	N participants	YFL	95% CI	Weight
EPIPORTO	324	−10.7	[−17.0; −4.5]	4.7%
SAGE Mexico	502	−4.2	[−15.2; 6.8]	2.7%
SEBAS	514	−9.3	[−13.1; −5.5]	6.0%
CRELES Pre 1945	871	−0.8	[−12.1; 10.5]	2.6%
NHANES 1999	882	−9.7	[−13.7; −5.6]	5.8%
SAGE Russia	883	−1.8	[−8.5; 4.8]	4.5%
SAGE South Africa	886	−1.2	[−10.5; 8.1]	3.3%
WLSS	903	−11.4	[−17.1; −5.6]	4.9%
NHANES 2001	925	−12.4	[−16.4; −8.3]	5.8%
CRELES RC	975	−7.8	[−32.3; 16.7]	0.8%
NSHAP	1389	−12.8	[−19.5; −6.0]	4.4%
HAALSI	1642	−16.3	[−21.8; −10.8]	5.0%
GAZEL	1920	−5.9	[−13.4; 1.7]	4.0%
SAGE Ghana	2016	−16.1	[−29.0; −3.2]	2.2%
ELSA	2104	−18.3	[−22.0; −14.6]	6.0%
TILDA	2149	−4.4	[−6.9; −1.9]	6.5%
NHANES III	2270	−16.3	[−20.4; −12.2]	5.8%
HRS	2466	−6.1	[−16.6; 4.5]	2.9%
SAGE India	3150	−13.0	[−16.7; −9.2]	6.0%
WHITEHALL II	4315	−8.8	[−12.5; −5.2]	6.0%
SAGE China	4644	−1.4	[−5.3; 2.5]	5.9%
CONSTANCES	12374	−9.4	[−16.8; −2.0]	4.1%
**Pooled YFL**		**−9.5**	**[−11.8; −7.1]**	**100.0%**
**Prediction interval**			**[−19.0; 0.1]**	

$I^2 = 77\%$, $\tau^2 = 19.63$

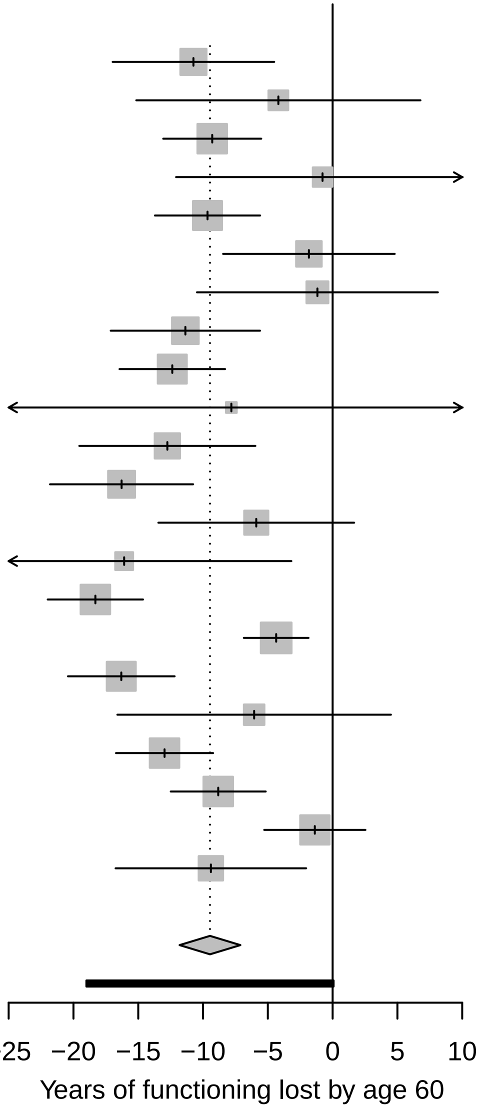

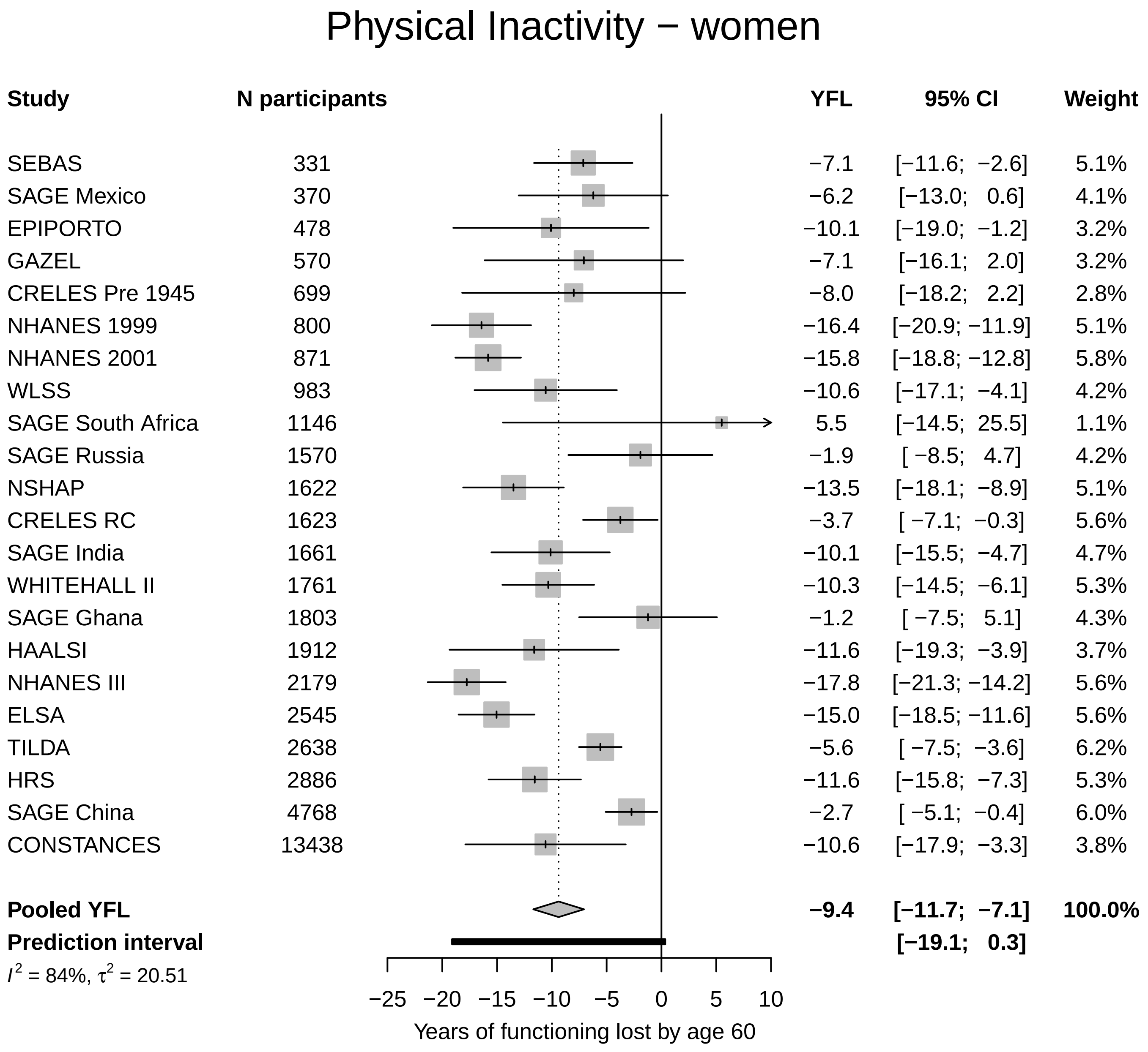
Physical Inactivity − women
Study
N participants
YFL
95% CI
Weight
SEBAS 331 −7.1 [−11.6; −2.6] 5.1%
SAGE Mexico 370 −6.2 [−13.0; 0.6] 4.1%
EPIPORTO 478 −10.1 [−19.0; −1.2] 3.2%
GAZEL 570 −7.1 [−16.1; 2.0] 3.2%
CRELES Pre 1945 699 −8.0 [−18.2; 2.2] 2.8%
NHANES 1999 800 −16.4 [−20.9; −11.9] 5.1%
NHANES 2001 871 −15.8 [−18.8; −12.8] 5.8%
WLSS 983 −10.6 [−17.1; −4.1] 4.2%
SAGE South Africa 1146 5.5 [−14.5; 25.5] 1.1%
SAGE Russia 1570 −1.9 [−8.5; 4.7] 4.2%
NSHAP 1622 −13.5 [−18.1; −8.9] 5.1%
CRELES RC 1623 −3.7 [−7.1; −0.3] 5.6%
SAGE India 1661 −10.1 [−15.5; −4.7] 4.7%
WHITEHALL II 1761 −10.3 [−14.5; −6.1] 5.3%
SAGE Ghana 1803 −1.2 [−7.5; 5.1] 4.3%
HAALSI 1912 −11.6 [−19.3; −3.9] 3.7%
NHANES III 2179 −17.8 [−21.3; −14.2] 5.6%
ELSA 2545 −15.0 [−18.5; −11.6] 5.6%
TILDA 2638 −5.6 [−7.5; −3.6] 6.2%
HRS 2886 −11.6 [−15.8; −7.3] 5.3%
SAGE China 4768 −2.7 [−5.1; −0.4] 6.0%
CONSTANCES 13438 −10.6 [−17.9; −3.3] 3.8%
Pooled YFL −9.4 [−11.7; −7.1] 100.0%
Prediction interval [−19.1; 0.3]
I² = 84%, τ² = 20.51
−25 −20 −15 −10 −5 0 5 10
Years of functioning lost by age 60
